# Exploring food choices among educated adults in the United Arab Emirates

**DOI:** 10.3389/fnut.2025.1708017

**Published:** 2026-01-12

**Authors:** Souzan Zidan, Serene Hilary, Carine Platat

**Affiliations:** 1 Department of Nutrition and Health, College of Medicine and Health Sciences, United Arab Emirates University, Al Ain, United Arab Emirates; 2 ASPIRE Research Institute for Food Security in the Drylands (ARIFSID), United Arab Emirates University, Al Ain, United Arab Emirates

**Keywords:** educated adults, food choice, food choice questionnaire, motives, sociodemographic characteristics, sustainability, UAE

## Abstract

**Introduction:**

Understanding food choice motives (FCMs) is crucial to designing dietary interventions to improve human health and environmental sustainability. This study aims to explore FCMs and to identify whether sociodemographic and anthropometric characteristics influence these motives among educated adults.

**Methods:**

This cross-sectional study surveyed 894 respondents between May 2023 and September 2024 at the United Arab Emirates University. The online questionnaire gathered self-reported information on sociodemographic characteristics, body weight and height, and self-reported knowledge of dietary guidelines. The FCMs were assessed using the adapted English and Arabic versions of the Food Choice Questionnaire (FCQ), covering nine domains: convenience, mood, health, natural content, familiarity, weight control, eco-ethics and price. Mann–Whitney U and Kruskal-Wallis tests were used to compare differences in food choice motivation among educated adults with different sociodemographic and anthropometric characteristics. Further analysis was done using linear regression to determine the association between sociodemographic and anthropometric characteristics (age, BMI, sex, nationality, marital status, family size, educational level, employment status, monthly income, diet counselling, nutrition education, and knowledge of dietary guidelines) and FCMs.

**Results:**

This study found that sensory appeal was the most important motive. In contrast, eco-ethics and familiarity were ranked as the least important. There was a statistically significant difference in FCMs among educated adults according to their sociodemographic and anthropometric characteristics (*p* < 0.05). Overall, only a small proportion of FCMs were affected by participants’ characteristics (3.9–12.6%). Sex was most often linked with variations in FCMs (i.e., all FCMs except for price were significantly more important to females than males; *p* < 0.05).

**Conclusion:**

Multiple motivations could determine people’s food choices. Eco-ethics and familiarity were the least important motives, suggesting that environmental and ethical concerns are not prioritized in food decisions. Our results also indicate that educated adults might be open to trying novel foods, which may support the introduction of more sustainable food choices. Moreover, sociodemographic and anthropometric factors had a limited role in influencing FCMs among educated adults, with the largest differences observed between males and females. Our findings also suggest that future dietary interventions targeting educated adults should be tailored to their sensory preferences.

## 1 Introduction

Food choice is a complex process and is not influenced only by sensory properties of the food (taste, odor, texture) but also by non-sensory properties such as emotional, biological, cultural, environmental, health, social, and economic factors (1–3). Individuals often prioritize multiple motives when selecting foods, and these priorities vary considerably between people (4–7). Such inter-individual variations can explain differences in dietary patterns. For instance, individuals with greater concerns about health, natural content, or ethics are more likely to consume healthy diets (diets rich in fruits and vegetables and limited in red meat). In contrast, individuals were more willing to consume unhealthy diets (e.g., Western diets) when they gave importance to familiarity, price, and convenience motives (5, 7, 8).

Earlier studies have also indicated that dietary patterns vary between individuals according to their demographic characteristics (9, 10). Some studies have found that affluent people are more likely to select healthy foods in comparison to disadvantaged people (11). Similarly, dietary habits are affected by sex, and results found that females consume more vegetables and fruits than males (11). However, little is known about the variation in food choice motives (FCMs) between these subgroups. Former studies found that women valued weight control and health motives more than men (6, 7, 12–14). Moreover, older individuals perceived health, natural content, and ethical concerns as more important than younger individuals (6, 12, 14).

Several comprehensive models have been developed to understand the reasons behind food selection. For example, Frust et al. (15) assembled the motives of food choice into three main components (life course, influences and personal systems). Each component is related to the others in such a way that it creates the pathway leading to the point of choice. Such a model was successful in describing the multifaceted nature of food choice; however, the comprehensive nature of this model hinders its ability to explain general food choice decisions (16). In contrast, the Food Choice Questionnaire (FCQ) has been widely recognized for its capability to explain general food choice based on its determinants (12). Originally, this questionnaire covered nine domains, including sensory properties of food, convenience, mood, health, natural content, familiarity, weight control, ethical concern, and price. Since its development, FCQ has been used to identify general food choice determinants in several populations around the world, including Australia (17), Canada (18), Greece (19), Finland (20), Ukraine (21), Uruguay (22), and the USA (13).

Over the past few decades, suboptimal diets have become a leading risk factor for mortality and disability-adjusted life years (DALYs) worldwide (23). Moreover, contemporary dietary patterns, particularly Western diets, are widely acknowledged as environmentally unsustainable and as key drivers of accelerating ecological degradation (24). Similar trends are observed across the Middle East and North Africa (MENA) region, including the United Arab Emirates (UAE), where the nutrition transition has led to a shift from traditional diets toward Westernized eating patterns high in refined sugars, saturated fats, and processed foods (25–27). This explains, in part, the increased prevalence of diet-related diseases such as diabetes, obesity, and cardiovascular diseases in the UAE. Furthermore, the country’s current food systems are unsustainable, characterized by high greenhouse gas emissions, excessive freshwater use, and intensive nitrogen and phosphorus use (28). In this context, food choices directly influence both human health and environmental sustainability; therefore, understanding what shapes people’s eating behavior may help researchers and public health professionals plan effective interventions to promote sustainable and healthy diets in the UAE (29, 30).

To our knowledge, previous studies examining FCMs in the UAE and the broader Middle East region remain scarce. More specifically, existing studies among Arab populations have not used the FCQ to systematically assess the full spectrum of FCMs, including sustainability- related motives, among adults, (31–33) nor have they examined their associations with anthropometric characteristics (33). Furthermore, only one study in the UAE employed the original FCQ; however, it was restricted to Emirati females and did not measure sustainability-related motives (34). Therefore, this is the first study in the UAE to include both sexes as well as non-Emirati residents, providing a more diverse and representative sample of adults, and the first in the region to assess sustainability-related food choice motives using the FCQ. Based on these considerations, the objective of the current study was to explore the FCMs, including sustainability-related motives, among a sample of educated adults in a university community in the UAE using the adapted Arabic and English versions of the FCQ. This study also examines whether these food choice motivations differ between individuals according to sociodemographic (e.g., sex, age, marital status, household size, education, and monthly income) and anthropometric characteristics.

## 2 Methods

### 2.1 Participants and study design

This cross-sectional study was conducted at the United Arab Emirates University (UAEU) among the university staff and student community between May 2023 and September 2024. Ethical approval was granted by the Social Sciences Research Ethics Committee at the UAEU (approval codes: ERSC_2023_2576 on 28.03.2023 and ERSC_2024_4357 on 21.02.2024), and the research was conducted in accordance with the Declaration of Helsinki. Informed consent was obtained from all participants after providing detailed information about the study’s purpose, risks, and benefits. The sample size was determined using Cochran’s formula with a 95% confidence interval, 5% significance level (0.05) and a marginal error of 0.05, with the correction for a finite population (35). The minimum sample size required for the study was 379. The eligibility criteria were participants from the UAEU community aged 18 years and above, willing to participate in the study and provide consent. A structured questionnaire in English and Arabic was developed in Qualtrics (see Supplementary Tables 1, 2). The study employed voluntary convenience sampling, whereby the survey link was shared with the university community via social media platforms in which the survey link was shared with the university community via social media and outreach emails. Participants were also randomly approached on campus at different times during weekdays. The first page of the questionnaire enclosed an information sheet explaining the purpose of the research. The participants needed about 15 min to complete the questionnaire. Clinical trial number: not applicable.

### 2.2 Measurements

Food motives were assessed using adapted English and Arabic versions of the FCQ after being validated among educated adults in the UAEU population. Details on the adaptation process will be reported in another publication (unpublished data). Briefly, the original English FCQ was revised to better align with the cultural context of the UAE population. Specifically, the original FCQ consists of 36 items covering nine domains: sensory appeal, convenience, mood, health, natural content, familiarity, weight control, ethical concern and price (12). During the adaptation process, item 20 from the ethical concern domain (“*Comes from countries I approve of politically”*) was removed as it was irrelevant to our research objectives. Then, five items were added to measure sustainability as proposed by Lindeman and Väänänen (20) and Share and Stewart-Knox (36); (*‘Has been produced in a way that animals’ rights have been respected*’), (*‘Has been prepared in an environmentally friendly way*’), (*‘Has been produced in a way which has not shaken the balance of nature*’), (‘*Is organic*’) and (‘*Has not been transported an excessive distance*’). This yielded an initial 40-item Arabic FCQ with ten domains (the original nine plus ecological welfare). After revising the English FCQ for cultural adherence, the tool was translated into Arabic by two professional translators. Then, two bilingual nutrition experts translated the Arabic questionnaire back into English. The Arabic FCQ’s comprehension and acceptability were tested among 33 Arabic-speaking adults. Afterwards, it was validated in a larger sample. During validation, the confirmatory factor analysis showed that the model with ten domains was marginally acceptable, whereas an alternative model with nine domains was more acceptable (Chi-square = 1293.09, df = 584, *p* < 0.05, X^2^/df = 2.21, RMSEA = 0.06). In the latter model, ethical concern and ecological welfare were merged into a single eco-ethics domain to capture sustainability-oriented motives better, and three items were also removed due to redundancy. The final validated model, therefore, comprises 37 items across nine domains: (i) health; (ii) mood; (iii) convenience; (iv) eco-ethics; (v) sensory appeal; (vi) familiarity; (vii) natural content; (viii) price; and (ix) weight control. The overall questionnaire demonstrated an excellent internal consistency, as reflected by McDonald’s omega (*Ω*) at 0.934 and Cronbach’s *α* at 0.934. Additionally, the intraclass correlation coefficient (ICC) values ranged from 0.868 to 0.955, indicating strong reliability and stability. Participants were asked to rate the importance of each item on a four-point Likert scale with 1 = “not at all important,” 2 = “a little important,” 3 = “moderately important” and 4 = “very important.” The mean score for each domain for each respondent was calculated based on their response codes.

The sociodemographic variables measured were sex, age, nationality, marital status, educational status, employment status, family size, and monthly income. Nationality was categorized into UAE nationals and expatriate residents due to the heterogeneity and small subgroup sizes within the expatriate population. Participants were also asked whether they had ever received a diet consultation or had formal nutritional education. They were also asked to rate their familiarity with dietary guidelines about healthy diets on a four-point scale (*“I have never heard of them,” “I have heard about them but know very little about them,” “I know a fair amount about them,” “I know a lot about them”*). Body Mass Index (BMI) was calculated from the self-reported body weight (kg) and height (cm), which previous research has shown to be a valid method (37), and then classified into four weight categories according to WHO cut-off points; underweight (BMI < 18.5 kg/ m^2^), normal weight (BMI 18.5–24.9 kg/ m^2^), overweight (BMI 25–29.9 kg/ m^2^), and obese (BMI > 30 kg/ m^2^) (38).

### 2.3 Statistical analyses

Data analyses were conducted using the statistical package for the social sciences SPSS® version 29.0 (IBM Corporation, Armonk, NY, USA). For descriptive and non-parametric statistical analysis, age was categorized into three groups (18–25, 26–45, and >45 years) based on age distribution in the sample. Continuous variables were summarized using means and standard deviations (SD), and categorical variables were summarized using proportions. The Chi-Square test was used to analyze the association between sex and categorical data. Fisher’s exact test was used when any expected cell count was fewer than five. Then, the Cramer’s V and the *Phi* coefficient were calculated to estimate the effect size (39).

Multiple response analysis was used to rank the different FCMs in the population. The proportion of the highest score (4 = ‘Very important’) in each food choice motive was used as the ranking criterion. Additionally, FCMs were ranked for their order of importance based on the median of the mean scores for each FCQ domain. Shapiro–Wilk tests indicated that the sociodemographic and anthropometric data were not normally distributed; consequently, the Mann–Whitney U test (for two groups) and the Kruskal-Wallis tests (for more than two groups) were used to evaluate the differences in importance of the motives by participants’ characteristics. Because sociodemographic variables were unevenly distributed in our sample, mean ranks were used rather than medians. Adjustments for multiple comparisons were performed using the Bonferroni correction. When significance was detected, the effect size (rank biserial correlation (r_rb_) for the Mann–Whitney U test, and the eta squared ($\eta_{H}^{2}$) for Kruskal-Wallis test) was calculated to measure the magnitude of differences in FCMs according to participants’ characteristics (39).

Linear regression analyses were conducted using the enter method, with a food choice motive as the outcome and sociodemographic and anthropometric characteristics as the exposure. The final model contained age (continuous), BMI (continuous), sex (female, male), nationality (UAE national, expatriate resident), marital status (single, married), family size (4 or less members, 5 or more members), educational level (high school, undergraduate, postgraduate), employment status (student/unemployed, employed), monthly income (less than AED 5000, AED 5001 – AED 20000, more than AED 20000), diet counselling (yes, no), nutrition education (yes, no), and self-reported knowledge of dietary guidelines (I have never heard about them, I have heard about them but know very little, I know a fair amount, I know a lot). Multicollinearity was determined using the variance inflation factor (VIF) value >10 in linear regression (40) (see Supplementary Table 3). The assumptions of the linear regression models were also tested using residuals. The normality of residuals was assessed using histograms, and residuals were plotted against fitted values and model variables. These diagnostics indicated no substantial violations of linear regression assumptions (please refer to Supplementary Figures 1, 2). For significant regression coefficients in the final models, effect sizes were calculated as Cohen’s f^2^ (39). All statistical tests were two-sided, and a *p*-value lower than 0.05 was considered significant.

## 3 Results

### 3.1 Participants’ characteristics

A total of 910 participants responded to the survey after giving their consent. However, sixteen participants who provided atypical responses to the FCQ were excluded. Specifically, these participants rated all 37 FCQ items with the maximum score (“4 = very important”), resulting in a total score of 148. Such uniform response patterns were considered outliers, as they suggest a lack of discrimination across FCMs. Therefore, the final sample consisted of 894 participants included in the analysis. The adequacy of the achieved sample was also evaluated using G*Power for a linear regression model with 12 predictors. The analysis (df = 881) yielded a noncentrality parameter *δ* = 5.98 and a critical t = 1.65, corresponding to an achieved power of 0.9 at *α* = 0.05 and a medium effect size (f^2^ = 0.15). This indicates an extremely high probability of detecting the observed effect, far exceeding the conventional 0.80 benchmark. The mean age was 23.4 ± 6.7 years with an age range of 18–62 years, of which 77.4% were females (Table 1). Most of the study population were Emirati (66.0%), single (89.3%), and belonged to large families (family of five members or more, 79.9%). A major proportion of the population were unemployed/ students (85.2%), a combined category that includes both students and unemployed individuals, reflecting the university setting of the study, while 14.8% were employed, representing faculty and university staff. Moreover, almost half of the study population (49.2%) had a high school education and a monthly income of less than AED 5000 (56.3%). The mean BMI for participants was 24.8 ± 7.9 kg/m^2^. The prevalence of underweight, normal weight, overweight and obese individuals was 12.0, 47.2, 24.7, and 16.1%, respectively. Nearly 31.1% have received at least one diet consultation, and around half of them (46.4%) have received some form of formal nutrition education. Furthermore, 58.4% of them self-reported knowing a fair amount about healthy eating guidelines (Table 1).

**Table 1:** Participants’ characteristics.

Variable		Total (*n* = 894)	
		Number (*n*)	Percentage (%)
Age	18–25 years old	695	77.7
	26–45 years old	187	20.9
	>45 years old	12	1.3
Sex	Female	692	77.4
	Male	202	22.6
Nationality	UAE national	590	66.0
	Expatriate resident	304	34.0
Marital status	Single	798	89.3
	Married	96	10.7
Family size	4 or less members	180	20.1
	5 or more members	714	79.9
Educational level	High school	440	49.2
	Undergraduate	291	32.6
	Postgraduate	163	18.2
Employment status	Unemployed/student	132	85.2
	Employed	762	14.8
Monthly income, AED	Less than 5,000	503	56.3
	5,001–20,000	206	23.0
	More than 20,000	185	20.7
Diet counseling	No	616	68.9
	Yes	278	31.1
Nutrition education	No	479	53.6
	Yes	415	46.4
Knowledge of dietary guidelines	I have never heard about them	39	4.4
	I have heard about them but know very little	199	22.3
	I know a fair amount	479	53.6
	I know a lot	177	19.8
BMI categories	Underweight	107	12.0
	Normal weight	422	47.2
	Overweight	221	24.7
	Obesity	144	16.1

AED: Emirati Dirham. “Unemployed/Students” represents a combined category including both unemployed individuals and students.

### 3.2 Motives of daily food choices

**Figure 1:**
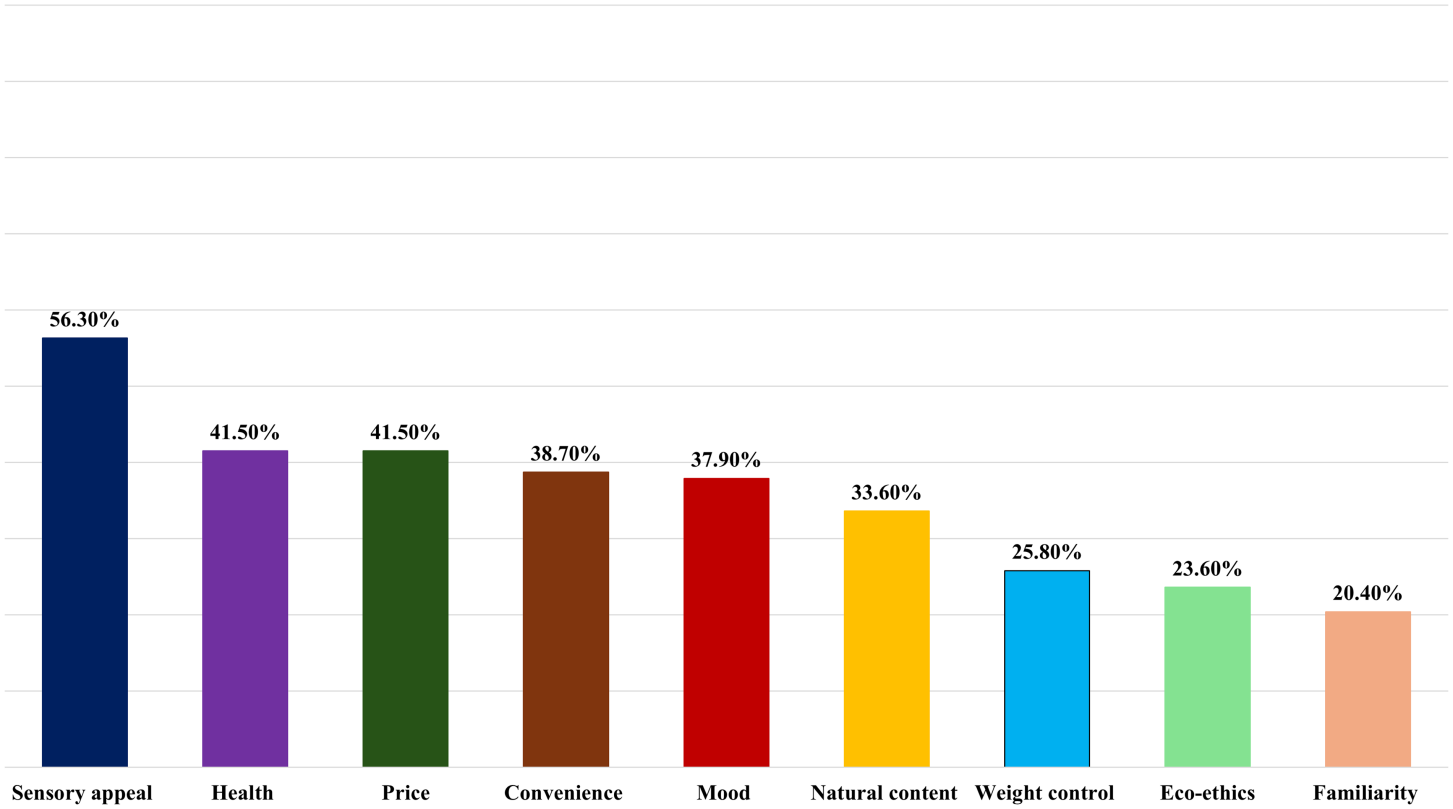
Ranking of food choice motives for the study participants. The proportion of the highest score (4 = ‘Very important’) in each domain is used as a criterion for ranking the food choice motives.

In the sample, sensory appeal (56.3%) was the strongest motive for daily food selection. Subsequently, the importance of food choice was similarly attributed to price (41.5%) and health (41.5%). Afterwards, convenience (38.7%) ranked third. However, eco-ethics (23.6%) and familiarity (20.4%) motives were the least important to the present participants (Figure 1). To provide a more comprehensive overview, we also calculated the median of the mean score for each FCQ domain. The median scores showed a similar trend: sensory appeal had the highest median (3.5), followed by price (3.3), health (3.2), and convenience (3.2). The lowest median scores were observed for eco-ethics (2.67) and familiarity (2.67) (Table 2).

**Table 2:** The median score for the mean scores for each food choice motive.

Food choice motives	Median score
Sensory appeal	3.50
Price	3.33
Health	3.20
Convenience	3.20
Mood	3.00
Natural content	3.00
Weight control	3.00
Eco-ethics	2.67
Familiarity	2.67

Table 3 shows that several reasons guided the participants’ food choices. Within the context of sensory appeal motivation, 73.9% of participants stated that food taste is very important. In the health domain, 53.6% of participants emphasized the importance of eating foods that keep them healthy. Regarding price motivation, 52.3% of participants preferred foods that offered good value for money. Within the convenience domain, ease of availability (42.8%) and cooking simplicity (42.5%) were the most frequently rated influential factors. Additionally, 55.7% of participants reported emotional satisfaction from food, affirming that “*Food cheers me up*.” Furthermore, 36.5% of participants considered natural ingredients the most important attribute when selecting food. The participants also expressed an interest in maintaining their weight through dietary choices by 37.5%, Within the eco-ethics domain, approximately 30.1% of participants rated the item “has been produced in a way that animals’ rights have been respected” as the most important. They also expressed concerns about the environment, particularly regarding animal rights in food production. Moreover, 29.4% of participants rated the item “the food I usually eat” as the most important within the familiarity domain.

**Table 3:** The most important food choice motives for the study participants^a^ (*n* = 894).

Food choice motive factors	Items	*n* (%)
Sensory appeal	Tastes good	**661** **(73.9)**
	Smells nice	507 (56.7)
	Has a pleasant texture	423 (47.3)
	Looks nice	423 (47.3)
Health	Keeps me healthy	**479 (53.6)**
	Is good for my skin/teeth/hair/nails etc.	446 (49.9)
	Is high in protein	375 (41.9)
	Contains a lot of vitamins and minerals	333 (37.2)
	Is high in fiber and roughage	221 (24.7)
Price	Is good value for money	**468 (52.3)**
	Is not expensive	340 (38.0)
	Is cheap	305 (34.1)
Convenience	Is easily available in shops and supermarkets	**383 (42.8)**
	Can be cooked very simply	**380 (42.5)**
	Can be bought in shops close to where I live or work	370 (41.4)
	Is easy to prepare	304 (34.0)
	Takes no time to prepare	295 (33.0)
Mood	Cheers me up	**498 (55.7)**
	Makes me feel good	449 (50.2)
	Helps me cope with stress	311 (34.8)
	Helps me relax	311 (34.8)
	Helps me to cope with life	288 (32.2)
	Keeps me awake/alert	175 (19.6)
Natural content	Contains natural ingredients	**326 (36.5)**
	Contains no artificial ingredients	274 (30.6)
Weight control	Helps me control my weight	**335 (37.5)**
	Is low in fat	226 (25.3)
	Is low in calories	132 (14.8)
Eco-ethics	Has been produced in a way that animals’ rights have been respected	**269 (30.1)**
	Has been produced in a way which has not shaken the balance of nature	251 (28.1)
	Has the country of origin clearly marked	234 (26.2)
	Is packaged in an environmentally friendly way	209 (23.4)
	Is organic	154 (17.2)
	Has not been transported an excessive distance	150 (16.8)
Familiarity	Is what I usually eat	**263 (29.4)**
	Is familiar	154 (17.2)
	Is like the food I ate when I was a child	131 (14.7)

a Based on the proportion of the highest score (4 = ‘Very important’). Bold values indicate the most frequently rated “very important” items within each domain.

### 3.3 The role of sociodemographic and anthropometric characteristics in shaping FCMs

Variations in food motives scores were most often associated with sex; that is, sex contributed to eight out of the nine models, followed by monthly income, age, knowledge of dietary guidelines, BMI, nationality, employment status, marital status, family size, educational level, and diet counselling. The characteristics included in the model only explained between 3.7 and 12.6% of the difference in food motives; that is, the selected factors account for 6.5% of sensory appeal, 9.3% of health, 3.7% of price, 7.0% of convenience, 10.5% of mood, 10.7% of natural content, weight control 12.6, 7.8% of eco-ethics, and 6.2% of familiarity (see Tables 4–6).

**Table 4:** The effect of different sociodemographic and anthropometric characteristics on sensory appeal, health, and price motive factors.

Variable		Sensory appeal (R^2^ = 0.065) *p*-value < 0.001^*^ ES = 0.07			Health (R^2^ = 0.093) *p*-value < 0.001^*^ ES = 0.10			Price (R^2^ = 0.037) *p*-value = 0.006^*^ ES = 0.04		
		B^a^	95% CI	*p*-value	B^a^	95% CI	*p*-value	B^a^	95% CI	*p*-value
Age		0.011	0.001, 0.020	0.037^*^	0.012	0.000, 0.023	0.043^*^	0.007	−0.005, 0.018	0.272
Sex	Female	0.247	0.148, 0.346	<0.001^*^	0.230	0.117, 0.344	<0.001^*^	0.086	−0.031, 0.203	0.148
	Male (ref.)									
Nationality	UAE national	0.086	−0.016, 0.188	0.099	−0.058	−0.175, 0.060	0.335	−0.132	−0.253, −0.011	0.032^*^
	Expatriate resident (ref.)									
Marital status	Single	−0.137	−0.306, 0.031	0.111	0.120	−0.074, 0.314	0.227	0.074	−0.126, 0.273	0.470
	Married (ref.)									
Family size	4 or less members (ref.)									
	5 or more members	0.160	0.049, 0.271	0.005^*^	−0.016	−0.144, 0.112	0.811	−0.042	−0.173, 0.090	0.536
Educational level	High school	−0.022	−0.182, 0.138	0.785	−0.320	−0.216, 0.152	0.735	0.100	−0.090, 0.289	0.302
	Undergraduate	−0.002	−0.150, 0.153	0.983	−0.018	−0.192, 0.156	0.841	0.064	−0.115, 0.243	0.486
	Postgraduate (ref.)									
Employment status	Unemployed/student	−0.027	−0.170, 0.117	0.716	−0.113	−0.279, 0.052	0.180	−0.023	−0.193, 0.148	0.795
	Employed (ref.)									
Monthly income, AED	Less than 5,000	0.072	−0.032, 0.177	0.176	0.068	−0.053, 0.189	0.269	0.172	0.048, 0.296	0.007^*^
	5,001–20,000	0.013	−0.112, 0.137	0.839	0.004	−0.139, 0.148	0.951	0.257	0.110, 0.404	<0.001^*^
	More than 20,000 (ref.)									
Diet counseling	No	0.015	−0.081, 0.112	0.758	−0.081	−0.192, 0.030	0.151	0.045	−0.069, 0.160	0.435
	Yes (ref.)									
Nutrition education	No	0.034	−0.056, 0.125	0.458	−0.046	−0.150, 0.058	0.387	−0.019	−0.126, 0.089	0.733
	Yes (ref.)									
Knowledge of dietary guidelines	I have never heard about them (ref.)									
	I have heard about them but know very little	−0.084	−0.291, 0.123	0.425	0.042	−0.196, 0.281	0.729	0.015	−0.230, 0.260	0.906
	I know a fair amount	−0.009	−0.207, 0.190	0.933	0.334	0.105, 0.563	0.004^*^	0.055	−0.181, 0.209	0.648
	I know a lot	−0.125	−0.340, 0.090	0.254	0.307	0.059, 0.554	0.015^*^	0.000	−0.254, 0.255	0.997
BMI		−0.003	−0.008, 0.002	0.191	−0.003	−0.008, 0.003	0.388	0.006	0.000, 0.012	0.048^*^

^*^Significance set as p-value ≤ 0.05.

a B values are unstandardized regression coefficients.

**Table 5:** The effect of different sociodemographic and anthropometric characteristics on convenience, mood, and natural content motive factors.

Variable		Convenience (R^2^ = 0.070) *p*-value < 0.001^*^ ES = 0.08			Mood (R^2^ = 0.105) *p*-value < 0.001^*^ ES = 0.18			Natural content (R^2^ = 0.107) *p*-value < 0.001^*^ ES = 0.12		
		B^a^	95% CI	*p*-value	B^a^	95% CI	*p*-value	B^a^	95% CI	*p*-value
Age		0.007	−0.003, 0.018	0.174	0.003	−0.008, 0.014	0.605	0.017	0.005, 0.030	0.008^*^
Sex	Female	0.317	0.211, 0.424	<0.001^*^	0.427	0.315, 0.540	<0.001^*^	0.217	0.089, 0.344	<0.001^*^
	Male (ref.)									
Nationality	UAE national	−0.013	−0.124, 0.097	0.813	−0.074	−0.190, 0.043	0.214	−0.150	−0.282, −0.018	0.026^*^
	Expatriate resident (ref.)									
Marital status	Single	0.159	−0.024, 0.341	0.088	−0.038	−0.231, 0.154	0.695	0.049	−0.169, 0.268	0.658
	Married (ref.)									
Family size	4 or less members (ref.)									
	5 or more members	−0.030	−0.150, 0.091	0.629	0.110	−0.017, 0.237	0.090	0.054	−0.090, 0.198	0.461
Educational level	High school	0.029	−0.144, 0.202	0.741	0.084	−0.099, 0.266	0.369	−0.112	−0.319, 0.095	0.290
	Undergraduate	−0.013	−0.176, 0.151	0.880	0.106	−0.067, 0.278	0.229	0.036	−0.232, 0.160	0.721
	Postgraduate (ref.)									
Employment status	Unemployed/student	−0.098	−0.254, 0.057	0.215	−0.083	−0.247, 0.081	0.321	−0.185	−0.372, 0.001	0.051
	Employed (ref.)									
Monthly income, AED	Less than 5,000	0.229	0.116, 0.342	<0.001^*^	0.243	0.123, 0.362	<0.001^*^	0.131	−0.004, 0.267	0.058
	5,001–20,000	0.199	0.065, 0.334	0.004^*^	0.202	0.060, 0.344	0.005^*^	0.023	−0.138, 0.184	0.779
	More than 20,000 (ref.)									
Diet counseling	No	−0.053	−0.157, 0.052	0.323	−0.022	−0.132, 0.088	0.700	−0.081	−0.206, 0.044	0.206
	Yes (ref.)									
Nutrition education	No	−0.045	−0.143, 0.053	0.365	−0.016	−0.119, 0.088	0.765	−0.073	−0.190, 0.045	0.225
	Yes (ref.)									
Knowledge of dietary guidelines	I have never heard about them (ref.)									
	I have heard about them but know very little	0.041	−0.265, 0.183	0.719	0.077	−0.159, 0.314	0.520	0.181	−0.087, 0.450	0.185
	I know a fair amount	−0.004	−0.220, 0.211	0.968	0.196	−0.031, 0.423	0.090	0.388	0.130, 0.646	0.003^*^
	I know a lot	−0.038	−0.270, 0.195	0.750	0.058	−0.187, 0.304	0.641	0.311	0.032, 0.590	0.029^*^
BMI		0.002	−0.004, 0.007	0.542	−0.001	−0.006, 0.005	0.819	−0.004	−0.011, 0.002	0.219

^*^Significance set as *p*-value ≤ 0.05.

a B values are unstandardized regression coefficients.

**Table 6:** The effect of different sociodemographic and anthropometric characteristics on weight control, eco-ethics, and familiarity motive factors.

Variable		Weight control (R^2^ = 0.126) *p*-value < 0.001^*^ ES = 0.15			Eco-ethics (R^2^ = 0.078) *p*-value < 0.001^*^ ES = 0.09			Familiarity (R^2^ = 0.062) *p*-value < 0.001^*^ ES = 0.07		
		B^a^	95% CI	*p*-value	B^a^	95% CI	*p*-value	B^a^	95% CI	*p*-value
Age		0.021	0.009, 0.034	0.001^*^	0.009	−0.003, 0.022	0.154	0.006	−0.006, 0.018	0.135
Sex	Female	0.174	0.046, 0.302	0.008^*^	0.369	0.241, 0.497	<0.001^*^	0.287	0.163, 0.410	<0.001^*^
	Male (ref.)									
Nationality	UAE national	−0.058	−0.190, 0.074	0.390	−0.113	−0.246, 0.019	0.093	−0.086	−0.214, 0.041	0.184
	Expatriate resident (ref.)									
Marital status	Single	0.310	0.091, 0.528	0.006^*^	0.020	−0.199, 0.239	0.860	−0.058	−0.269, 0.153	0.591
	Married (ref.)									
Family size	4 or less members (ref.)									
	5 or more members	0.042	−0.102, 0.187	0.564	0.014	−0.131, 0.158	0.854	0.121	−0.018, 0.260	0.089
Educational level	High school	−0.087	−0.294, 0.121	0.413	0.079	−0.129, 0.287	0.456	0.113	−0.087, 0.313	0.269
	Undergraduate	−0.037	−0.233, 0.159	0.712	0.108	−0.088, 0.305	0.279	0.130	−0.059, 0.319	0.177
	Postgraduate (ref.)									
Employment status	Unemployed/student	−0.214	−0.401, −0.028	0.024^*^	−0.175	−0.362, 0.012	0.066	−0.062	−0.242, 0.118	0.500
	Employed (ref.)									
Monthly income, AED	Less than 5,000	0.165	0.029, 0.301	0.017^*^	0.158	0.022, 0.294	0.023^*^	0.296	0.156, 0.427	<0.001^*^
	5,001–20,000	0.058	−0.103, 0.220	0.478	0.057	−0.105, 0.218	0.490	0.173	0.017, 0.329	0.029^*^
	More than 20,000 (ref.)									
Diet counseling	No	−0.242	−0.367, −0.117	<0.001^*^	−0.037	−0.162, 0.088	0.565	0.043	−0.078, 0.163	0.486
	Yes (ref.)									
Nutrition education	No	−0.073	−0.190, 0.044	0.223	−0.036	−0.154, 0.081	0.544	−0.018	−0.132, 0.095	0.750
	Yes (ref.)									
Knowledge of dietary guidelines	I have never heard about them (ref.)									
	I have heard about them but know very little	0.240	−0.029, 0.509	0.080	0.109	−0.160, 0.387	0.426	0.045	−0.214, 0.304	0.734
	I know a fair amount	0.429	0.171, 0.687	0.001^*^	0.293	0.034, 0.551	0.026^*^	0.101	−0.148, 0.350	0.426
	I know a lot	0.370	0.091, 0.649	0.009^*^	0.178	−0.101, 0.457	0.212	−0.021	−0.290, 0.248	0.877
BMI		0.009	0.003, 0.016	0.005^*^	−0.001	−0.007, 0.006	0.847	0.000	−0.006, 0.007	0.905

^*^Significance set as *p*-value ≤ 0.05.

a B values are unstandardized regression coefficients.

#### 3.3.1 FCQ domain: sensory appeal

Regression analysis showed that sensory appeal was significantly associated with age, sex, and family size, with a small effect size (Cohen’s f^2^ = 0.07). The regression coefficients in Table 4 showed that with each increase in age, the importance of sensory appeal increased by 0.011 (95% CI 0.001, 0.020). Likewise, females rated sensory appeal higher than males by an average of 0.247 (95% CI 0.148, 0.346) (see Table 4). This finding was also supported by the Mann–Whitney test, which revealed significant differences between females and males for the sensory appeal (*p* < 0.001), with a small effect size (r_rb_: 0.19) (see Table 7). Table 4 revealed that participants with a family of five or more placed more value on sensory appeal by an average of 0.160 (95% CI 0.049, 0.271) than those with a family of four or fewer.

**Table 7:** Differences in the mean rank of food choice motives in relation to participants’ characteristics.

Variable	SA	HE	PR	CON	MD	NC	WC	ECO-EC	FAM
Age in years									
18–25 (*n* = 695)	442.15^a^	435.52^a^	438.22^a^	441.73^a^	448.01^a^	421.22^b^	428.29^b^	438.05^a^	449.78^a^
26–45 (*n* = 187)	458.54^a^	482.45^a^	471.79^a^	473.98^a^	445.85^a^	537.24^a^	510.07^a^	474.72^a^	435.11^a^
>45 (*n* = 12)	585.08^a^	596.88^a^	606.58^a^	368.79^a^	443.79^a^	571.21^ab^	585.29^ab^	570.54^a^	508.71^a^
ES	–	–	–	–	–	0.04	0.02	–	–
Sex									
Female (*n* = 692)	466.85^a^	472.59^a^	451.18^a^	476.30^a^	481.74^a^	465.63^a^	460.99^a^	474.50^a^	468.43^a^
Male (*n* = 202)	381.21^b^	361.54^b^	434.91^a^	348.82^b^	330.19^b^	385.40^b^	401.28^b^	355.01^b^	375.78^b^
ES	−0.19	−0.25	––	−0.29	−0.34	−0.18	−0.13	−0.27	−0.21
Nationality									
Emirati (*n* = 590)	455.75^a^	438.55^a^	426.39^b^	444.92^a^	446.41^a^	419.94^b^	432.66^b^	434.22^b^	443.19^a^
Expatriate resident (*n* = 304)	431.49^a^	464.87^a^	488.46^a^	452.50^a^	449.62^a^	501.00^a^	476.31^a^	473.27^a^	455.86^a^
ES	–	–	0.14	–	–	0.18	0.10	0.09	–
Marital status									
Single (*n* = 798)	442.95^a^	444.73^a^	444.55^a^	449.50^a^	447.44^a^	437.89^b^	444.62^a^	443.44^a^	446.80^a^
Married (*n* = 96)	485.32^a^	470.54^a^	471.99^a^	430.84^a^	448.02^a^	527.38^a^	471.42^a^	481.22^a^	453.32^a^
ES	–	–	–	–	–	0.20	–	–	–
Family size									
4 or less members (*n* = 180)	414.51^a^	454.06^a^	473.09^a^	452.03^a^	424.68^a^	469.39^a^	450.80^a^	456.06^a^	428.02^a^
5 or more members (*n* = 714)	455.82^a^	445.85^a^	441.05^a^	446.36^a^	453.25^a^	441.98^a^	446.67^a^	445.34^a^	452.41^a^
ES	–	–	–	–	–	–	–	–	–
Employment status									
Employed (*n* = 132)	463.24^a^	490.91^a^	475.96^a^	454.91^a^	438.31^a^	539.30^a^	533.33^a^	487.52^a^	445.35^a^
Unemployed/student (*n* = 762)	444.77^a^	439.98^b^	442.57^a^	446.22^a^	449.09^a^	431.60^b^	432.63^b^	440.57^a^	447.87^a^
ES	––	0.11	–	–	–	0.24	0.23	–	–
Educational level									
High school (*n* = 440)	433.37^a^	421.58^b^	437.18^a^	438.10^a^	435.85^a^	402.94^c^	411.37^b^	423.84^a^	437.37^a^
Undergraduate (*n* = 291)	462.54^a^	463.40^ab^	443.86^a^	451.69^a^	468.60^a^	463.99^b^	464.51^a^	467.81^a^	466.91^a^
Postgraduate (*n* = 163)	458.81^a^	489.08^a^	481.85^a^	465.38^a^	441.27^a^	538.36^a^	514.68^a^	475.11^a^	440.21^a^
ES	–	0.01	–	–	–	0.04	0.02	–	–
Monthly income									
Less than AED 5000 (*n* = 503)	459.80^a^	451.04^a^	447.60^b^	467.38^a^	473.52^a^	445.73^a^	449.50^a^	460.07^a^	475.88^a^
AED 5001 – AED 20000 (*n* = 206)	434.80^a^	451.28^a^	500.42^a^	459.06^a^	443.70^a^	470.19^a^	459.77^a^	447.99^a^	436.46^ab^
More than AED 20000 (*n* = 185)	428.20^a^	433.67^a^	388.30^c^	380.59^b^	380.97^b^	427.05^a^	428.38^a^	412.79^a^	382.62^b^
ES	–	–	0.02	0.02	0.02	–	–	–	0.02
BMI categories									
Underweight (*n* = 107)	463.61^a^	437.28^a^	376.18^c^	429.16^a^	474.89^a^	424.25^a^	319.50^b^	426.57^a^	441.89^a^
Normal weight (*n* = 422)	444.65^a^	452.04^a^	446.30^abc^	442.12^a^	439.28^a^	452.59^a^	446.16^a^	461.14^a^	447.13^a^
Overweight (*n* = 221)	463.28^a^	456.25^a^	460.61^b^	468.21^a^	456.12^a^	462.73^a^	494.68^a^	452.82^a^	458.16^a^
Obese (*n* = 144)	419.67^a^	428.35^a^	483.90^ab^	445.11^a^	438.01^a^	426.47^a^	474.11^a^	414.93^a^	436.39^a^
ES	–	–	0.01	–	–	–	0.04	–	–
Knowledge of dietary guidelines									
I have never heard about them (*n* = 39)	471.64^a^	332.95^b^	426.00^a^	406.83^a^	385.12^ab^	323.96^b^	305.28^b^	351.58^b^	422.59^a^
I have about them but know very little (*n* = 199)	419.90^a^	359.22^cb^	437.41^a^	415.20^a^	412.99^b^	393.65^cb^	383.42^cb^	399.12^b^	439.84^a^
I know a fair amount (*n* = 479)	465.26^a^	477.06^a^	456.13^a^	459.98^a^	473.51^a^	470.26^a^	471.32^a^	474.81^a^	462.55^a^
I know a lot (*n* = 177)	425.14^a^	492.00^a^	440.22^a^	459.01^a^	429.66^ab^	473.67^a^	486.41^a^	449.12^ab^	420.88^a^
ES	–	0.05	–	–	0.01	0.02	0.03	0.02	–
Diet consultation									
Yes (*n* = 278)	439.15^a^	484.08^a^	435.94^a^	473.41^a^	457.91^a^	473.17^a^	522.46^a^	463.30^a^	442.31^a^
No (*n* = 616)	451.27^a^	430.99^b^	452.72^a^	435.81^b^	442.80^a^	435.91^b^	413.67^b^	440.37^a^	449.84^a^
ES	––	−0.12	–	−0.09	–	−0.08	−0.24	–	–
Nutrition education									
Yes (*n* = 415)	437.31^a^	474.15^a^	444.22^a^	464.00^a^	455.99^a^	469.60^a^	484.78^a^	461.15^a^	447.59^a^
No (*n* = 479)	456.33^a^	424.41^b^	450.34^a^	433.20^a^	440.14^a^	428.35^b^	415.20^b^	435.68^a^	447.42^a^
ES	–	−0.11	–	–	–	−0.09	−0.16	–	–

SA: sensory appeal; HE: health; CON: convenience; MD: mood; NC: natural content; WC: weight control; ECO-EC: eco-ethics; FAM: familiarity ES: effect size. Significance set as *p*-value ≤ 0.05. Values that share a superscript letter are not significantly different from each other according to the multiple comparison test (*p* < 0.05). Significance values have been adjusted by the Bonferroni correction for multiple tests.

#### 3.3.2 FCQ domain: health

Age, sex, and knowledge of dietary guidelines were positively associated with the health domain, with a small effect size (Cohen’s f^2^ = 0.10). The older the participants were, the more they valued health motive by an average of 0.012 (95% CI 0.000, 0.023). Females rated health motive higher than males by an average of 0.230 (95% CI 0.117, 0.344). Also, participants who know a fair amount about dietary guidelines and those who know a lot about dietary guidelines rated health motives as more important compared to those who have never heard about dietary guidelines, with 0.334 (95%CI 0.105, 0.563) and 0.307 (95% CI 0.059, 0.554), respectively (see Table 4).

The non-parametric tests were also consistent with the previous findings. For instance, the Mann–Whitney test showed significant differences between females and males for the health (*p <* 0.001), with a medium effect size (r_rb_: 0.25) (see Table 7). Additionally, the Kruskal-Wallis H test showed that different knowledge levels about dietary guidelines had significant differences in health (*p* < 0.001), with a small effect size ($\eta_{H}^{2}$: 0.05). According to pairwise comparisons, participants who had never heard about nutrition guidelines paid less attention to health than those who knew a fair amount about dietary guidelines. Moreover, those who know very little about dietary guidelines placed less importance on health than those who know a fair amount. Besides that, we found that those who reported high knowledge of dietary guidelines gave higher importance scores to health than those who had never heard of or knew very little about dietary guidelines (see Table 7).

Besides these findings, our analysis also revealed that employed participants significantly value health more than unemployed/ students (*p* < 0.036), with a small effect size (r_rb_: 0.11) (see Table 7). Furthermore, the results of the Kruskal-Wallis H test showed that participants with different education levels attributed different scores on health (*p* = 0.007), with a small effect size ($\eta_{H}^{2}$: 0.01). Pairwise comparisons showed that participants with postgraduate degrees had a significantly higher mean rank value for health than those with high school degrees, and that participants with postgraduate degrees gave higher importance scores to health than those with high school degrees (see Table 7).

Lastly, the Mann–Whitney test revealed that individuals who received diet consultation (*p* = 0.004, r_rb_: 0.12) and those with formal nutrition education (*p =* 0.004, r_rb_: 0.11) paid significantly more attention to health motive when making their food choices in comparison to their counterparts who did not receive diet consultation and those who did not receive formal nutrition education, with a small effect size (see Table 7).

#### 3.3.3 FCQ domain: price

Regression analysis indicated that price was significantly associated with nationality, monthly income, and BMI, with a small effect size (Cohen’s f^2^ = 0.04). As illustrated in Table 4, UAE nationals rated the price motive significantly less important than expatriate residents by −0.132 (95% CI -0.253, −0.011). Another finding from Table 4 is that participants with a monthly income less than AED 5000 and those with a monthly income ranging from AED 5001 to AED 20000 rated price more important than those with a monthly income more than AED 20000 by 0.172 (95% CI 0.048, 0.296) and 0.257 (95%CI 0.110, 0.404), respectively. Furthermore, the importance of price increased by 0.006 (95% CI 0.000, 0.012) with each increase in BMI (see Table 4).

In accordance with regression analysis, the Mann–Whitney test confirmed that Emirati participants gave significantly lower ratings for price (*p* < 0.001), with a small effect size (r_rb_: 0.14) (see Table 7). The Kruskal-Wallis H test also showed that monthly income has a significant effect on price (*p* < 0.001), with a $\eta_{H}^{2}$ of 0.02, indicating a small effect size. Pairwise comparison showed that the mean rank score for price was significantly lower among participants with a monthly income of more than AED 20000 compared to those with a monthly income in the range of AED 5001 to AED 20000 or lower. Surprisingly, those with a monthly income less than AED 5000 gave lower importance to price when selecting their food than participants with a monthly income ranging from AED 5001 to AED 20000 (see Table 7).

The analysis again revealed significant differences in price (*p* = 0.008) among individuals with different BMI, with a small effect size ($\eta_{H}^{2}$: 0.01). More specifically, underweight individuals rated the price motives significantly less than those who were overweight or obese (see Table 7).

#### 3.3.4 FCQ domain: convenience

The regression model showed that convenience was significantly associated with sex and monthly income, with a small effect size (Cohen’s f^2^ = 0.08). By looking at Table 5, females rated convenience as more important than males by a factor of 0.317 (95% CI 0.211, 0.424). Moreover, participants with a monthly income of less than AED 5000 and those with a monthly income ranging from AED 5001 to AED 20000 rated convenience more important than those with a monthly income of more than AED 20000 by 0.229 (95% CI 0.116, 0.342) and 0.199 (95% CI 0.065, 0.334), respectively (see Table 5).

The Mann–Whitney test showed that females significantly rated convenience higher than males (*p* < 0.001), with a small effect size (r_rb_: 0.29) (see Table 7). Additionally, individuals who received diet consultation paid more attention to convenience (*p* = 0.043) when making their food choices in comparison to those who did not receive diet consultation, with a small effect size (r_rb_: 0.09) (see Table 7).

The results of the Kruskal-Wallis H test also confirmed that monthly income has a significant effect on convenience (*p* < 0.001), with a$\;\eta_{H}^{2}$ of 0.02, indicating a small effect size. Specifically, pairwise comparison showed that the mean rank score for convenience was significantly lower among participants with a monthly income of more than AED 20000 compared to those with a monthly income in the range of AED 5001 to AED 20000 or lower (see Table 7).

#### 3.3.5 FCQ domain: mood

Sociodemographic characteristics that were significantly associated with mood were sex and monthly income, with a medium effect size (Cohen’s f^2^ = 0.18). The regression coefficients indicated that females rated mood higher than males by an average of 0.427 (95% CI 0.315, 0.540). Moreover, people with a monthly income less than AED 5000 and those with a monthly income ranging from AED 5001 to AED 20000 rated the importance of mood 0.243 (95% CI 0.123, 0.362) and 0.202 (95% CI 0.060, 0.344), respectively, higher than those with a monthly income more than AED 20000 (see Table 5).

These results were supported by non-parametric analysis. The Mann–Whitney test indicated a significant difference between females and males in mood scores (*p* < 0.005), with a medium effect size (r_rb_: 0.34) (see Table 7). The Kruskal-Wallis H test further confirmed that monthly income has a significant effect on mood (*p* < 0.001), with a $\eta_{H}^{2}$ of 0.02, indicating a small effect size. Specifically, pairwise comparisons showed that participants with a monthly income of more than AED 20000 had significantly lower mood scores compared to those with a monthly income in the range of AED 5001 to AED 20000 or lower (see Table 7).

In addition, the analysis showed that different knowledge levels about dietary guidelines had significant differences in mood (*p* = 0.009), with a small effect size ($\eta_{H}^{2}$: 0.01). According to pairwise comparisons, participants who know very little about dietary guidelines gave less importance to mood than those who know a fair amount about dietary guidelines (see Table 7).

#### 3.3.6 FCQ domain: natural content

Four factors were significantly associated with natural content: age, sex, nationality, and knowledge of dietary guidelines, with a small effect size (Cohen’s f^2^ = 0.12). According to Table 5, with every increase in age, the importance of natural content increased by 0.017 (95% CI 0.005, 0.030). Likewise, females cared more about food naturalness than males by an average of 0.217 (95% CI 0.089, 0.344). Also, UAE nationals rated food naturalness significantly less important than expatriate residents by −0.150 (95% CI -0.282, −0.018). In addition, those who know a fair amount about dietary guidelines and those who know a lot about dietary guidelines rated natural content more important compared to those who have never heard about dietary guidelines by 0.388 (95% CI 0.130, 0.646) and 0.311 (95% CI 0.032, 0.590), respectively (see Table 5).

Consistent with regression analysis, the Kruskal-Wallis H test showed that the 26–45 age group scored significantly higher on natural content motive than the 18–25 age group with a small effect size (*p* < 0.001,$\;\eta_{H}^{2}$: 0.04). Similarly, the results of the Mann–Whitney test showed that females placed significantly more importance on natural content than males (*p* < 0.005), with a small effect size (r_rb_: 0.18) (see Table 7). Nationality differences were also evident, as Emirati participants gave significantly lower ratings for natural content (*p* < 0.001), with a small effect size (r_rb_: 0.18) (see Table 7).

In addition, married participants rated natural content (*p* < 0.001) more than single participants, with an effect size of 0.20, which represents a small effect (see Table 7). Employment status also had a significant effect on natural content (*p* < 0.001), with a small effect size (r_rb_: 0.24). The results showed that employed participants valued natural content more than unemployed/ students (see Table 7).

The results of the Kruskal-Wallis H test also showed that educational attainment was significantly associated with different scores on natural content (*p* < 0.001), with a small effect size ($\eta_{H}^{2}$: 0.04). Pairwise comparisons indicated that participants with a postgraduate degree had a significantly higher mean rank value on natural content than those with a high school education or undergraduate qualification. Findings also indicated that participants with high school degrees gave lower importance scores for natural content than undergraduates and postgraduates (see Table 7).

The analysis again showed that different knowledge levels about dietary guidelines had significant differences in natural content (*p* < 0.001), with a small effect size ($\eta_{H}^{2}$: 0.02). According to pairwise comparisons, participants who had never heard of nutrition guidelines paid less attention to natural content than those who knew a fair amount about dietary guidelines. Moreover, those who know very little about dietary guidelines placed less importance on natural content than those who know a fair amount. Besides that, we found that those who reported high knowledge of dietary guidelines gave higher importance scores to natural content than those who had never heard of or knew very little about dietary guidelines (see Table 7).

Lastly, the Mann–Whitney test revealed that individuals who received diet consultation (*p* = 0.042, r_rb_: 0.08) and those with formal nutrition education (*p =* 0. 015, r_rb_: 0. 09) paid significantly more attention to natural content motive when making their food choices in comparison to their counterparts who did not receive diet consultation and those who did not receive formal nutrition education, with a small effect size (see Table 7).

#### 3.3.7 FCQ domain: weight control

Regression analysis revealed that weight control was significantly associated with age, sex, marital status, employment status, monthly income, diet counselling, knowledge of dietary guidelines, and BMI, with a medium effect size (Cohen’s f^2^ = 0.15). More precisely, the regression coefficients showed that with every increase in age, the importance of weight control increased by 0.021 (95% CI 0.009, 0.034) (see Table 6). Additionally, females rated weight control higher than males by an average of 0.174 (95%CI 0.046, 0.302). Likewise, single participants reported greater weight control concerns than their married counterparts, by an average of 0.310 (95% CI 0.091, 0.528). Furthermore, students and unemployed participants placed less importance on weight control −0.214 (95% CI -0.401, −0.028) compared to employers. Besides that, those with a monthly income less than AED 5000 rated weight control more important by an average of 0.165 (95% CI 0.029, 0.301) compared to those with a monthly income higher than AED 20000. Moreover, participants who have not consulted with dieticians placed less importance on weight control by −0.242 (95% CI -0.367, −0.117) than those who have not consulted with dieticians. Table 6 also indicated that those who know a fair amount about dietary guidelines and those who know a lot about dietary guidelines rated weight control as more important compared to those who have never heard about dietary guidelines by 0.429 (95% CI 0.171, 0.687) and 0.370 (95% CI 0.091, 0.649), respectively. Finally, with every increase in BMI, the importance of weight control increased by 0.009 (95% CI 0.003, 0.016) (see Table 6).

Non-parametric tests supported some of these findings. For instance, the Kruskal-Wallis H test showed that the 26–45 age group scored significantly higher on weight control motives than the 18–25 age group, with a small effect size (*p* < 0.001,$\;\eta_{H}^{2}$: 0.02). The Mann–Whitney test also showed significant differences between females and males for the weight control motive with a small effect size (p < 0.001, r_rb_: 0.13) (see Table 7). Furthermore, the results confirmed that employed participants valued weight control significantly more than unemployed/ students, with a small effect size (*p* < 0.001, r_rb_: 0.23) (see Table 7). The analysis also showed significant differences in weight control among individuals with different BMIs, with a small effect size (*p* < 0.001, $\eta_{H}^{2}$: 0.04) More specifically, underweight individuals rated weight control motives significantly less than those who were overweight or obese. Besides that, we found that the mean rank score for weight control motive was considerably lower among underweight participants in contrast to normal weight participants (see Table 7).

In addition, the analysis showed that differences in knowledge of dietary guidelines were associated with significant differences in weight control, with a small effect size (*p* < 0.001, $\eta_{H}^{2}$: 0.03). According to pairwise comparisons, participants who had never heard of nutrition guidelines paid less attention to weight control than those who knew a fair amount about dietary guidelines. Moreover, those who know very little about dietary guidelines placed less importance on weight control than those who know a fair amount. Besides that, we found that those who reported high knowledge of dietary guidelines gave higher weight-control importance scores than those who had never heard of or knew very little about dietary guidelines (see Table 7). Individuals who received diet consultation paid more attention to weight control when making their food choices in comparison to those who did not receive diet consultation, with a small effect size (*p* < 0.001, r_rb_: 0.24) (see Table 7).

Besides these findings, Emirati participants gave significantly lower ratings for weight control with a small effect size (*p* = 0.016, r_rb_: 0.10) (see Table 7). The results of the Kruskal-Wallis H test also showed that participants with different education levels attributed different scores on weight control with a small effect size (*p* < 0.001, $\eta_{H}^{2}$: 0.02). Pairwise comparisons showed that participants with high school degrees gave lower importance scores for weight control than undergraduates and postgraduates (see Table 7). Moreover, the Mann–Whitney test revealed that those who received formal nutrition education scored significantly higher on weight control in contrast to their counterparts who did not receive formal nutrition education, with a small effect size (*p* < 0.001, r_rb_: 0.16) (see Table 7).

#### 3.3.8 FCQ domain: eco-ethics

Eco-ethics was significantly associated with sex, monthly income and knowledge of dietary guidelines, with a small effect size (Cohen’s f^2^ = 0.09). More precisely, females rated eco-ethics higher than males by an average of 0.369 (95% CI 0.241, 0.497). Likewise, participants with a monthly income less than AED 5000 rated eco-ethics more important than those with a monthly income more than AED 20000 by 0.158 (95% CI 0.022, 0.294). Also, those who knew a fair amount about dietary guidelines rated eco-ethics more important compared to those who had never heard about dietary guidelines by 0.293 (95% CI 0.034, 0.551) (see Table 6).

The results of the Mann–Whitney test revealed sex-based differences in FCMs, with females assigning greater importance to eco-ethics (*p* < 0.001), with a small effect size (r_rb_ = 0.27). Emiratis also rated eco-ethics significantly lower than non-Emiratis (*p* = 0.032) with a small effect size (r_rb_ = 0.09). In addition, the analysis showed that knowledge of dietary guidelines was significantly associated with eco-ethics (*p* < 0.001) with small effect sizes (*η*^2^_h_ = 0.02). Pairwise comparisons indicated that participants with no or limited knowledge of dietary guidelines gave lower importance to eco-ethics compared to those with a fair amount of knowledge about dietary guidelines (see Table 7).

#### 3.3.9 FCQ domain: familiarity

Lastly, two factors were significantly associated with familiarity, that is, sex and monthly income, with a small effect size (Cohen’s f^2^ = 0.07). Table 6 shows that females rated familiarity higher than males by an average of 0.287 (95% CI 0.163, 0.410). In addition, participants with a monthly income less than AED 5000 and those with a monthly income ranging from AED 5001 to AED 20000 rated familiarity more important than those with a monthly income more than AED 20000 by 0.296 (95% CI 0.156, 0.427) and 0.173 (95% CI 0.017, 0.329), respectively (see Table 6).

These findings are consistent with non-parametric analysis. Specifically, the Mann–Whitney test confirmed that females rated familiarity significantly higher than males (*p* < 0.001), with a small effect size (r_rb_: 0.21) (see Table 7). The results of the Kruskal-Wallis H test also showed that monthly income has a significant effect on familiarity (*p* < 0.001), with a $\eta_{H}^{2}$ of 0.02, indicating a small effect size. Pairwise comparisons showed that those with a monthly income of more than AED 20000 placed less importance on food familiarity when making food choices than those with a monthly income of less than AED 5000 (see Table 7).

## 4 Discussion

### 4.1 Motives of daily food choices

The overall findings showed that sensory appeal, followed by non-sensory factors such as price, health, and convenience, were the population’s strongest FCMs. This is consistent with former studies by Vorage et al. (41), Carrillo et al. (42), Honkanen and Frewer (43), and Steptoe et al. (12). These authors found that sensory appeal was the most important motive in food selection. This implies that individuals are affected mainly by food’s smell, taste, flavour, and appearance when making their food choices. According to Deliens and his colleagues, young adulthood is a stage in which individuals become more responsible for their food choices. In this life period, young adults have to pay more attention to their food prices (44). In addition, young adults, especially those living in student residences, may face new challenges, including high academic stress, sleep difficulties, and a lack of time to prepare meals. Altogether, all of these factors may explain the high tendency of young adults to select inexpensive and convenient foods (45).

According to our results, eco-ethics and familiarity were rated as the bottom two domains of FCMs. This trend aligns with findings from European countries and Great Britain (46, 47). A cross-sectional study of Belgians (*n* = 458), Hungarians (*n* = 401), Romanians (*n* = 229), and Filipinos (*n* = 332) reported familiarity and ethical concern as the least influential motives. However, among Belgians, mood was rated as less important than ethical concern (46). Similarly, the Food4Me study found that familiarity and ethical concern were consistently ranked as the least important FCMs across nine European countries (Germany, Greece, Ireland, Poland, Portugal, Spain, the Netherlands, the UK and Norway) (47). A study by Dikmen et al. (48) found that ethical concerns and weight control were unimportant for Turkish consumers when consuming food.

Simply, our results might indicate that participants are willing to try novel foods and dishes. This aligns with a recent study showing that 51.2% of Emirati consumers in the UAE are open to alternative protein (49). According to Monin and Szczurek (50), people are rewarded and encouraged to look for variety and investigate novel things. Another possible explanation for this finding is the multicultural nature of the UAE, where expatriates outnumber nationals (51). This cultural diversity exposes residents to a wide range of culinary traditions, which may reduce the salience of familiarity as a food choice motive and foster the acceptance of novel foods. Notably, even among Emiratis, previous research has shown a tendency to adopt new foods, suggesting that cultural differences may be less pronounced among young populations (34). Indeed, globalization facilitated people’s access to distinct types of food. Consequently, people become more interested and enthusiastic to try new foods (52). Nevertheless, as the FCQ does not capture behavioral intentions or tendencies such as food neophobia or openness to novelty, additional research employing instruments that directly assess willingness to try novel foods is required to substantiate this interpretation.

Additionally, it seems that our participants do not consider ethical concerns (e.g.*, is packaged in an environmentally friendly way, has the country of origin clearly marked*) and ecological welfare (e.g.*, has been produced in a way that animals’ rights have been respected, has been produced in a way which has not shaken the balance of nature, is organic, has not been transported an excessive distance*) when they are making their food choices, suggesting a limited awareness of the environmental impact of food choices Recent studies in the UAE showed that the country of origin is generally not an important factor in food preferences for either Emirati or expatriate consumers (53, 54). Another study in the UAE highlights a gap between consumers’ positive attitudes toward sustainable food packaging and their actual purchasing behavior (31).

Conversely, a former study also highlighted a growing trend in organic food consumption in the UAE, driven primarily by health and environmental awareness. However, the study also identified key barriers to organic food consumption, including high cost, limited availability, short shelf life, taste, and inadequate knowledge (55). In our study, participants were educated adults, and nearly half of them demonstrated a fair level of knowledge of dietary guidelines; nevertheless, this did not necessarily translate into greater awareness of organic food. These findings suggest that even among an educated population with a moderate knowledge of dietary guidelines, significant gaps remain in specific awareness and perceptions related to organic food. This highlights the complexity of factors influencing organic food consumption and the need for targeted interventions that go beyond general nutrition education to address these barriers and enhance both awareness and accessibility of organic food products.

The analysis of individual items demonstrated the primary priorities of our participants when making their food choices. Participants mostly valued taste, surpassing other sensory attributes such as appearance, smell, and texture. This finding is in agreement with previous research indicating that while these attributes contribute to overall food perception, their influence remains secondary to taste (56). Prescott further highlighted that even when foods appear visually appealing or smell pleasant, it is unlikely to be regularly consumed if the taste is not preferred (56). Additionally, neuroscientific evidence suggests that taste activates the brain’s reward centres more strongly than smell or texture, reinforcing preference and repeated consumption (57). In addition, participants predominantly acknowledged the significance of consuming foods that maintain health, reflecting an increasing awareness of the link between diet and overall well-being. This finding aligns with a recent study from the UAE, which reported that participants largely agreed on the importance of eating food beneficial to health (32). Furthermore, participants highly emphasized the importance of food offering good value for money, depicting the perceived balance between cost and quality in food choices. This finding aligns with prior research by Stewart et al. (58), which showed that price elasticity affects food purchasing decisions, with consumers tending to favor cost-effective options that also meet their taste and nutritional preferences.

Our findings also showed that the ease of food availability in supermarkets was an important motive influencing participants’ food choices. A recent systematic review found that the availability and positioning of food items in retail environments significantly impact purchasing decisions and dietary intake among adult (59). Besides that, our findings indicate that participants prioritize foods that can be easily cooked. This preference is particularly pronounced among individuals with busy lifestyles or limited cooking skills. Our finding here complements a previous finding showing that individuals with lower cooking confidence are more likely to opt for convenience cooking products, which require less preparation effort (60).

### 4.2 The role of sociodemographic characteristics in shaping FCMs

We found that participants in the 18–25 age group valued “long-term-oriented” motives (health, natural content, and weight control) less than those in the 26–45 age group and those older than 45, which was reported by earlier studies (61, 62). For example, Du and his colleagues found that participants in the 18–25 age group gave lower ratings for long-term-oriented motives compared to adults aged 26 years and older (61). In contrast, a study from Australia showed that weight control is more important among young emerging adults (aged 17–20) than older emerging adults (aged 21–29) (41). One possible explanation for our finding is that health problems become more common with increasing age, which may, in turn, result in more long-term orientation in middle-aged and older adults (43). Additionally, weight gain concerns may increase with age, making older individuals more conscious of weight control. For instance, a previous study reported that middle-aged individuals were more dissatisfied with their body shape and underestimated their actual weight more than younger adults (63). Also, we observed that the older the participants were, the more they cared about the sensory appeal of food. This finding contradicts previous research, which showed that younger individuals give more importance to “short-term-oriented” motives (e.g., sensory appeal) compared to older individuals (62). However, it should be noted that in our sample, participants aged 45 and older represent a very small subgroup compared to the other age categories; therefore, the significant differences observed may reflect limited cell sizes rather than actual population effects.

In addition, findings reported here demonstrate that females placed higher importance on all FCMs except for price compared with males. Similarly, Schliemann et al.’s study (2019) found that females rated convenience, health, mood, and sensory appeal higher than males (14). Konttinen and his colleagues also revealed that females ranked convenience, ethical concern, health, mood, natural content, sensory appeal, as well as weight control as more important in their food choice compared to males (7). Our findings provide an indication that females still have the primary task for shopping and cooking in many families, and this explains why females are more involved and preoccupied with food-related decisions (64). Another probable interpretation is that females are conscious of the health repercussions of food, and more aware of their body weight, and eating healthy eating habits (e.g., consuming fruits and vegetables) (65, 66). It should also be noted that these differences reflect emotional, social, and biological differences (67). However, in our study the effect sizes associated with these sex differences ranged from small to medium, indicating that the magnitude of these associations was modest. Accordingly, these findings should be interpreted with caution.

Studies exploring the association between nationality and FCMs are limited. As far as we know, this study was one of the few to compare the food motives of UAE nationals with those of expatriate residents and found that UAE nationals valued eco-ethics, price, natural content, and weight control less than expatriate residents. This indicates that UAE nationals are less concerned about ethical issues, ecological welfare, and food naturalness when selecting their foods. A previous study conducted in the UAE found that less than one-third of participants were knowledgeable about food additives (68). One possible reason why UAE nationals scored lower on eco-ethics could be a lack of understanding of how food consumption affects the environment. A study conducted at Zayed University reported that the primary barrier to adopting sustainable nutrition among UAE students was a lack of knowledge (69). Our observations also indicate that UAE nationals paid little attention to food prices. One possible interpretation is that 23.2% of UAE nationals in the current sample had a monthly income exceeding AED 20000, while 15.8% of expatriate residents had a monthly income exceeding AED 20000. Apart from this, the UAE government has restructured its social welfare program for low-income citizens, allocating AED 28 billion to support various allowances, including a food supplement subsidy. This subsidy aims to mitigate the impact of food price inflation by covering 75% of food price increases for eligible Emirati families, thereby helping them maintain a reasonable standard of living (70). As a result, Emirati citizens may be less aware of food prices compared to expatriates. Still, more studies are needed to directly examine the factors that shape the relationship between nationality and price-related food choices in the UAE.

In addition, our findings also depict that UAE nationals are less conscious about their weight (71). A recent study by Khair et al. (71) revealed that overweight or obese Emirati women were 13 times more likely to underestimate their weight status. In addition, these individuals were 3.6 times more likely to correctly identify their appropriate gestational weight gain, suggesting limited awareness about weight management needs among Emirati women.

There is little evidence of whether marital status influences FCMs. An Irish study demonstrated that married people rated natural content and weight control slightly higher than unmarried individuals (14). Another study by Konttinen et al. (62) observed a weak effect of marital status on FCMs. Moreover, some studies have explored the relationships between marital status and eating habits. For instance, a systematic review concluded that consumption of fruits and vegetables was higher among married people compared to single individuals (72). According to our study, married participants rated food naturalness more, while singles placed greater emphasis on weight control motives when making food choices.

The current findings also showed that employed participants were more concerned about health, natural content, and weight control than unemployed participants/ students. Employment status remains an under-researched factor in relation to FCMs. A recent work among Mexican adults found that employed participants attached less importance to sensory appeal and greater importance to food identity (73). Several factors probably drive these observations. On the one hand, employers may have greater financial means to buy natural food (e.g., organic foods) as well as weight loss products, given that such foods have been shown to cost more per calorie (74, 75) compared to students or unemployed individuals. On the other hand, employed individuals in a university setting are usually educated and may have higher nutrition literacy than unemployed individuals. However, in the real world, employment status does not necessarily correlate directly with education level, as employed individuals can range from highly educated professionals to those with limited formal education (e.g., daily wage laborers). Therefore, it is essential to distinguish between the university community – typically more educated - and the broader category of employed individuals to avoid oversimplifying the interpretation of the data.

Unsurprisingly, our findings indicated that participants with a higher educational level prioritized health, natural content, and weight control in their daily food selection more than participants with a high school degree. High educational levels may improve nutrition knowledge as well as encourage individuals to adopt healthy dietary patterns (76, 77). Besides that, Siu and his colleagues found that less-educated individuals are more likely to perceive weight control as expensive and require cooking skills (78). This is possible because these individuals have lower nutritional knowledge and less healthy cooking skills (78).

Another finding in the current study is that participants with high monthly incomes were less concerned about convenience, familiarity, mood, and price when making their food choices. Likewise, Konttinen e al. (62) revealed that people in the highest-income quartile cared less about familiarity than people in the lowest-income quartile. In addition, they found a weak effect of income on convenience and mood. According to the mechanisms of social distinction, people with high socioeconomic levels may have a greater desire to incorporate unfamiliar foods and dishes into their diets as a way to distinguish themselves from those with low socioeconomic levels (79, 80). Consistent with our findings, Konttinen et al. (62) also found monthly income to be one of the best predictors for price-cheap motives. Studies of low-income individuals indicated that price and food quality are significant motives in shopping decisions (81, 82). Similarly, Markovina et al. (47) revealed that individuals may prefer low prices over other preferences under financial hardship. A surprising finding in our study is that people with low monthly incomes considered weight control motives more important than their higher-income counterparts.

### 4.3 The role of anthropometric characteristics and familiarity with dietary guidelines in shaping FCMs

Consistent with former studies in other countries (14, 61, 83), we found that higher BMI was associated with greater concern about weight control when selecting food. This means that overweight and obese individuals have a greater desire to lose weight, and normal-weight individuals are more likely to maintain their body weight. Not surprisingly, overweight and obese individuals rated prices more important than underweight individuals. A similar trend has also been reported in a former study (14), which showed that price was more valued with each increase in BMI. As discussed earlier, weight-loss products are usually expensive (75), thus overweight and obese individuals are looking for affordable products that may help them lose or maintain their body weight.

In addition, we found that knowledge of dietary guidelines is associated with several motives, including eco-ethics, health, mood, natural content, and weight control. Previous studies have explored the association between nutritional knowledge and dietary behavior. For instance, Scalvedi et al. (84) found that the highest score of nutrition knowledge corresponds to the highest adherence to healthy eating habits.

### 4.4 Limitations and strengths

This study has a few limitations. Firstly, the anthropometric data were self-reported, which may lead to inaccurate BMI estimates. Secondly, the representativeness of our sample was limited by several demographic imbalances. Our sample included more females than males, reflecting the sex distribution of the UAEU community, where approximately 70% of students are female. Another explanation for this imbalance is that females are usually more concerned about nutrition topics (85), while males are less enthusiastic about participating in population-based surveys (86). However, the effect size for this gender imbalance was small, suggesting that the imbalance did not substantially bias the sample characteristics (see Supplementary Table 4). On the other hand, employed individuals and those aged 25 years or older were underrepresented in the current study, reflecting the university-based setting in which the research was conducted. Additionally, national statistics indicate that youth comprise over 50% of the UAE workforce, which further aligns with the demographic profile of our sample (87). Thirdly, our study used voluntary convenience sampling in a university setting, which may limit the representativeness of the sample and, therefore, the generalizability of the findings to the larger adult population in the UAE. Moreover, some subgroups in our sample were very small (e.g., participants aged 45 years and older), which reduces statistical power, increases the likelihood of unstable estimates, and may elevate the risk of Type I and Type II errors. These subgroup findings should therefore be interpreted with caution. Therefore, a nationwide study is highly needed to better understand food choices among the broader adult population in the UAE. Besides that, we recommend that future studies be done on a balanced sample to address the limitations of this study, such as the underrepresentation of males, employed individuals, and those over 25 years old. Furthermore, the actual eating behavior of the participants was not assessed. Another limitation is the cross-sectional design of this study, which does not allow for drawing causal inferences. Additionally, information on participants’ academic discipline was not collected, which may have influenced their FCMs. Another limitation is that the limited body of research on food choice motives in Arab countries constrained the extent of regional comparison and required reliance on international literature. Finally, although several predictors reached statistical significance, the regression models explained only a small proportion of the variance in FCMs. This limited explanatory power may suggest that additional unmeasured factors likely influence these motives. Therefore, the findings should be interpreted with caution, and future studies should include a broader range of predictors to better capture the complexity of food choice behavior. Despite these limitations, the present study provides interesting insights regarding the motives underlying food consumption among educated adults in the UAEU using a validated FCQ revised to fit that population. Another strength lies in the inclusion of both English and Arabic versions of the questionnaire, which enhanced accessibility for participants who were more comfortable with Arabic. Furthermore, unlike a former study from the UAE (34)The present study included both sexes as well as non-Emirati residents, resulting in a more diverse and representative sample. It also used multivariate analysis to study factors (e.g., socio-demographic and anthropometric characteristics) that might influence FCMs. Most importantly, this work extends beyond individual preferences by introducing an eco-ethical domain that links FCMs to environmentally sustainable dietary behaviors, offering a novel and timely contribution within the UAE context and the broader Middle East region.

### 4.5 Implications

Scholars and policymakers should take into account the sensory appeal of meals when designing dietary interventions aimed at benefiting human health, attaining sustainable development, as well as guiding food production. For example, interventions should prioritize enhancing the taste, aroma, and overall palatability of sustainable, healthy food choices. This could include culinary training workshops and the reformulation of institutional menus to make sustainable, healthy meals more flavorful and visually appealing.

Furthermore, sociodemographic and anthropometric characteristics such as age, sex, nationality, marital status, family size, educational level, employment status, monthly income, diet consultation, and BMI categories may be important to consider when designing an intervention. More specifically, it is essential to identify population profiles that would benefit from positive dietary changes or that demonstrate the greatest need for intervention. For example, females consistently placed greater importance on most motives, except for the price, suggesting that interventions targeting women may emphasize on the sensory appeal of food, convenience, health-related factors (e.g., health, natural content, weight control), as well as mood, familiarity, and eco-ethical considerations. Additionally, socioeconomic differences in FCMs also warrant attention. Individuals with lower income valued price and convenience more strongly, emphasizing the need for policies that improve the affordability and accessibility of nutritious foods, such as campus-based subsidies, price promotions, or collaborations with local vendors to offer low-cost, healthy, sustainable meals. Furthermore, participants who received diet counselling or formal nutrition education rated health and weight control motives higher, underscoring the value of personalized and interactive interventions. Offering accessible nutrition counselling services, particularly for males and individuals with limited knowledge, could enhance motivation for positive dietary change.

We also suggest quantitatively or qualitatively exploring FCMs before designing a dietary intervention. Future interventions should be tailored to people’s characteristics (e.g., sociodemographic and anthropometric).

## 5 Conclusion

Among educated adults from the UAEU community, sensory appeal emerged as the most important motive for food choices, whereas eco-ethics and familiarity were unimportant. This indicates that ecological welfare and ethical concerns are not currently prioritized in food selection. Consequently, future dietary interventions should focus on enhancing the taste and palatability of food, as these are the factors most likely to drive behavior change. The low importance of familiarity suggests that educated adults may be open to trying novel foods as long as they are tasty, which could be an opportunity to introduce more sustainable food choices without requiring prior exposure. Furthermore, the most significant differences in FCMs were observed between males and females. In the current study, knowledge of dietary guidelines did not play a key role in shaping food choices. The findings of this study may assist in designing dietary interventions for people with different sociodemographic and anthropometric characteristics; however, additional research is needed to explore whether FCMs translate into eating behavior.

## Data Availability

The raw data supporting the conclusions of this article will be made available by the authors, without undue reservation.

## References

[1] Prescott J Young O O'Neill LO Yau N Stevens R. Motives for food choice: a comparison of consumers from Japan, Taiwan, Malaysia and New Zealand. Food Qual Prefer. (2002) 13:489–95. doi: 10.1016/S0950-3293(02)00010-1

[2] McCarthy S. Weekly patterns, diet quality and energy balance. Physiol Behav. (2014) 134:55–9. doi: 10.1016/j.physbeh.2014.02.046, 24589367

[3] Bisogni CA Connors M Devine CM Sobal J. Who we are and how we eat: A qualitative study of identities in food choice. J Nutr Educ Behav. (2002) 34:128–39. doi: 10.1016/S1499-4046(06)60082-1, 12047837

[4] Steptoe A Wardle J. Motivational factors as mediators of socioeconomic variations in dietary intake patterns. Psychol Health. (1999) 14:391–402. doi: 10.1080/08870449908407336

[5] Pollard TM Steptoe A Wardle J. Motives underlying healthy eating: using the food choice questionnaire to explain variation in dietary intake. J Biosoc Sci. (1998) 30:165–79. doi: 10.1017/S0021932098001655 9746823

[6] Renner B Sproesser G Strohbach S Schupp HT. Why we eat what we eat. the eating motivation survey (TEMS). Appetite. (2012) 59:117–28. doi: 10.1016/j.appet.2012.04.004, 22521515

[7] Konttinen H Sarlio-Lähteenkorva S Silventoinen K Männistö S Haukkala A. Socio-economic disparities in the consumption of vegetables, fruit and energy-dense foods: the role of motive priorities. Public Health Nutr. (2013) 16:873–82. doi: 10.1017/S1368980012003540, 22857602 PMC10271369

[8] Allès B Péneau S Kesse-Guyot E Baudry J Hercberg S Méjean C. Food choice motives including sustainability during purchasing are associated with a healthy dietary pattern in French adults. Nutr J. (2017) 16:58. doi: 10.1186/s12937-017-0279-9, 28923107 PMC5604508

[9] Zagorsky JL Smith PK. The association between socioeconomic status and adult fast-food consumption in the US. Econ Hum Biol. (2017) 27:12–25. doi: 10.1016/j.ehb.2017.04.004 28472714

[10] Pechey R Monsivais P. Socioeconomic inequalities in the healthiness of food choices: exploring the contributions of food expenditures. Prev Med. (2016) 88:203–9. doi: 10.1016/j.ypmed.2016.04.012, 27095324 PMC4910945

[11] Health. Healthy Ireland survey 2017: summary of findings. Dublin, Ireland: Government Publications (2017).

[12] Steptoe A Pollard TM Wardle J. Development of a measure of the motives underlying the selection of food: the food choice questionnaire. Appetite. (1995) 25:267–84. doi: 10.1006/appe.1995.0061, 8746966

[13] Glanz K Basil M Maibach E Goldberg J Snyder DAN. Why Americans eat what they do: taste, nutrition, cost, convenience, and weight control concerns as influences on food consumption. J Am Diet Assoc. (1998) 98:1118–26. doi: 10.1016/S0002-8223(98)00260-0, 9787717

[14] Schliemann D Woodside JV Geaney F Cardwell C McKinley MC Perry I. Do socio-demographic and anthropometric characteristics predict food choice motives in an Irish working population? Br J Nutr. (2019) 122:111–9. doi: 10.1017/S0007114519000941, 31190657

[15] Furst T Connors M Bisogni CA Sobal J Falk LW. Food choice: a conceptual model of the process. Appetite. (1996) 26:247–66. doi: 10.1006/appe.1996.0019, 8800481

[16] Eertmans A Baeyens F Van den Bergh O. Food likes and their relative importance in human eating behavior: review and preliminary suggestions for health promotion. Health Educ Res. (2001) 16:443–56. doi: 10.1093/her/16.4.443, 11525391

[17] Lockie S Lyons K Lawrence G Mummery K. Eating ‘green’: motivations behind organic food consumption in Australia. Soc. Ruralis. (2002) 42:23–40. doi: 10.1111/1467-9523.00200

[18] Martins Y Pliner P. The development of the food motivation scale. Appetite. (1998) 30:94. doi: 10.1006/appe.1997.0127, 9515047

[19] Chryssochoidis G Krystallis A Perreas P. Ethnocentric beliefs and country-of-origin (COO) effect: impact of country, product and product attributes on Greek consumers' evaluation of food products. Eur J Mark. (2007) 41:1518–44. doi: 10.1108/03090560710821288

[20] Lindeman M Väänänen M. Measurement of ethical food choice motives. Appetite. (2000) 34:55–9. doi: 10.1006/appe.1999.0293, 10744892

[21] Biloukha OO Utermohlen V. Correlates of food consumption and perceptions of foods in an educated urban population in Ukraine. Food Qual Prefer. (2000) 11:475–85. doi: 10.1016/S0950-3293(00)00020-3

[22] Ares G Gámbaro A. Influence of gender, age and motives underlying food choice on perceived healthiness and willingness to try functional foods. Appetite. (2007) 49:148–58. doi: 10.1016/j.appet.2007.01.006, 17335938

[23] Afshin A Sur PJ Fay KA Cornaby L Ferrara G Salama JS . Health effects of dietary risks in 195 countries, 1990–2017: a systematic analysis for the global burden of disease study 2017. Lancet. (2019) 393:1958–72. doi: 10.1016/S0140-6736(19)30041-8, 30954305 PMC6899507

[24] Franco CC Rebolledo-Leiva R Gonzalez-Garcia S Feijoo G Moreira MT. Addressing the food, nutrition and environmental nexus: the role of socio-economic status in the nutritional and environmental sustainability dimensions of dietary patterns in Chile. J Clean Prod. (2022) 379:134723. doi: 10.1016/j.jclepro.2022.134723

[25] Johnston JL Fanzo JC Cogill B. Understanding sustainable diets: A descriptive analysis of the determinants and processes that influence diets and their impact on health, food security, and environmental sustainability. Adv Nutr. (2014) 5:418–29. doi: 10.3945/an.113.005553, 25022991 PMC4085190

[26] Ng SW Zaghloul S Ali H Harrison G Yeatts K El Sadig M . Nutrition transition in the United Arab Emirates. Eur J Clin Nutr. (2011) 65:1328–37. doi: 10.1038/ejcn.2011.135, 21772317 PMC3304306

[27] Popkin BM Adair LS Ng SW. Global nutrition transition and the pandemic of obesity in developing countries. Nutr Rev. (2012) 70:3–21. doi: 10.1111/j.1753-4887.2011.00456.x, 22221213 PMC3257829

[28] GNR Global nutrition report- United Arab Emirates: The burden of malnutrition at a glance. Bristol: Development Initiatives Poverty Research Ltd. (2021).

[29] Frehner A Van Zanten HHE Schader C De Boer IJM Pestoni G Rohrmann S . How food choices link sociodemographic and lifestyle factors with sustainability impacts. J Clean Prod. (2021) 300:126896. doi: 10.1016/j.jclepro.2021.126896

[30] Szalonka K Stańczyk E Gardocka-Jałowiec A Waniowski P Niemczyk A Gródek-Szostak Z. Food choices and their impact on health and environment. Energies. (2021) 14:5460. doi: 10.3390/en14175460

[31] Ajith A Nassar S. Sustainable food packaging: exploring the consumer attitude-behaviour gap in the UAE In: Economic and social development: Book of proceedings. Varazdin, Croatia: Varazdin Development and Entrepreneurship Agency. (2021). 173–81.

[32] Cheikh Ismail L Osaili TM Obaid RS Hashim M Ahmed M Al-Fayadh F . Food choice motivations and perceptions of healthy eating: a cross-sectional study among consumers in the UAE. BMC Public Health. (2025) 25:442. doi: 10.1186/s12889-024-20836-8, 39905339 PMC11792200

[33] Alharthi B Informing health-related behaviour change in Saudi Arabia: a social marketing approach. Newcastle upon Tyne, United Kingdom: Newcastle University. (2017).

[34] Haroun D Ehsanallah A Smail L. Motives of college students for choice of traditional food in the UAE. Br Food J. (2025) 127:2216–32. doi: 10.1108/BFJ-10-2024-1082

[35] Woolson RF Bean JA Rojas PB. Sample size for case-control studies using Cochran's statistic. Biometrics. (1986) 42:927–32. doi: 10.2307/2530706 3814733

[36] Share M Stewart-Knox B. Determinants of food choice in Irish adolescents. Food Qual Prefer. (2012) 25:57–62. doi: 10.1016/j.foodqual.2011.12.005

[37] Hodge JM Shah R McCullough ML Gapstur SM Patel AV. Validation of self-reported height and weight in a large, nationwide cohort of US adults. PLoS One. (2020) 15:e0231229. doi: 10.1371/journal.pone.0231229, 32282816 PMC7153869

[38] World Health Organization (WHO). Cut-off for BMI according to WHO standards. (2018). Available online at: https://gateway.euro.who.int/en/indicators/mn_survey_19-cut-off-for-bmi-according-to-who-standards/ (Accessed December 30, 2025).

[39] Cohen J. Statistical power analysis for the behavioral sciences. London: Routledge (2013).

[40] Senaviratna N A Cooray TMJ. Diagnosing multicollinearity of logistic regression model. Asian J Probab Stat. (2019) 5:1–9. doi: 10.9734/ajpas/2019/v5i230132

[41] Vorage L Wiseman N Graca J Harris N. The association of demographic characteristics and food choice motives with the consumption of functional foods in emerging adults. Nutrients. (2020) 12:2582. doi: 10.3390/nu12092582, 32854396 PMC7551355

[42] Carrillo E Varela P Salvador A Fiszman S. Main factors underlying consumers' food choice: a first step for the understanding of attitudes toward “healthy eating”. J Sens Stud. (2011) 26:85–95. doi: 10.1111/j.1745-459x.2010.00325.x

[43] Honkanen P Frewer L. Russian consumers' motives for food choice. Appetite. (2009) 52:363–71. doi: 10.1016/j.appet.2008.11.009, 19073227

[44] Deliens T Clarys P De Bourdeaudhuij I Deforche B. Determinants of eating behaviour in university students: a qualitative study using focus group discussions. BMC Public Health. (2014) 14:53. doi: 10.1186/1471-2458-14-53, 24438555 PMC3905922

[45] Marquis M. Exploring convenience orientation as a food motivation for college students living in residence halls. Int J Consum Stud. (2005) 29:55–63. doi: 10.1111/j.1470-6431.2005.00375.x

[46] Januszewska R Pieniak Z Verbeke W. Food choice questionnaire revisited in four countries. Does it still measure the same? Appetite. (2011) 57:94–8. doi: 10.1016/j.appet.2011.03.014, 21477629

[47] Markovina J Stewart-Knox BJ Rankin A Gibney M de Almeida MDV Fischer A . Food4Me study: validity and reliability of food choice questionnaire in 9 European countries. Food Qual Prefer. (2015) 45:26–32. doi: 10.1016/j.foodqual.2015.05.002

[48] Dikmen D İnan-Eroğlu E Göktaş Z Barut-Uyar B Karabulut E. Validation of a Turkish version of the food choice questionnaire. Food Qual Prefer. (2016) 52:81–6. doi: 10.1016/j.foodqual.2016.03.016

[49] Maqsood S Ajayi FF Mostafa H Lawal KG Mubaiwa J Alantali N . Are Emirati consumers in United Arab Emirates open to alternative proteins? Insights into their attitudes and willingness to replace animal protein sources. Front Sustain Food Syst. (2025) 9:1446790. doi: 10.3389/fsufs.2025.1446790

[50] Monin B Szczurek LM (2014) Food cultures

[51] Wikipedia. Demographics of the United Arab Emirates. (2024). Available online at: https://en.wikipedia.org/wiki/Demographics_of_the_United_Arab_Emirates (Accessed September 01, 2025).

[52] Pearcey SM Zhan GQ. A comparative study of American and Chinese college students’ motives for food choice. Appetite. (2018) 123:325–33. doi: 10.1016/j.appet.2018.01.011, 29337255

[53] McShane PE Sheaves M Fathelrahman E Maqsood S Degefa B Yousif NNMK . Consumer preferences for seafood inform aquaculture development in the United Arab Emirates. Aquaculture. (2025) 599:742105. doi: 10.1016/j.aquaculture.2024.742105

[54] Cheikh Ismail L Hashim M Osaili TM Faris ME Naja F Radwan H . Exploring sustainable food choices among adults in the United Arab Emirates: a cross-sectional study. Front Sustain Food Syst. (2023) 7:1307758. doi: 10.3389/fsufs.2023.1307758

[55] Al-Taie WAA Rahal MKM Al-Sudani ASA Al-Farsi KAO. Exploring the consumption of organic foods in the United Arab Emirates. SAGE Open. (2015) 5:2158244015592001. doi: 10.1177/2158244015592001

[56] Prescott J. Chemosensory learning and flavour: perception, preference and intake. Physiol Behav. (2012) 107:553–9. doi: 10.1016/j.physbeh.2012.04.008, 22525491

[57] Small DM Zatorre RJ Dagher A Evans AC Jones-Gotman M. Changes in brain activity related to eating chocolate: from pleasure to aversion. Brain. (2001) 124:1720–33. doi: 10.1093/brain/124.9.1720, 11522575

[58] Stewart H Blisard N Bhuyan S Nayga RM (2004). The demand for food away from home: full-service or fast food? Washington, DC, United States: U.S. Department of Agriculture, Economic Research Service (USDA-ERS). doi: 10.22004/ag.econ.33953

[59] Shaw SC Ntani G Baird J Vogel CA. A systematic review of the influences of food store product placement on dietary-related outcomes. Nutr Rev. (2020) 78:1030–45. doi: 10.1093/nutrit/nuaa024, 32483615 PMC7666915

[60] Brasington N Bucher T Beckett EL. Frequency of convenience cooking product use is associated with cooking confidence, creativity, and markers of vegetable intake. Nutrients. (2023) 15:966. doi: 10.3390/nu15040966, 36839322 PMC9967409

[61] Du T Luo C Gao Z Chang Y Zhuang X Ma G. Application and validation of a Chinese version of the food choice questionnaire (FCQ). Food Qual Prefer. (2024) 120:105260. doi: 10.1016/j.foodqual.2024.105260

[62] Konttinen H Halmesvaara O Fogelholm M Saarijärvi H Nevalainen J Erkkola M. Sociodemographic differences in motives for food selection: results from the LoCard cross-sectional survey. Int J Behav Nutr Phys Act. (2021) 18:71. doi: 10.1186/s12966-021-01139-2, 34078396 PMC8173871

[63] Bibiloni MM Coll JL Pich J Pons A Tur JA. Body image satisfaction and weight concerns among a Mediterranean adult population. BMC Public Health. (2017) 17:39. doi: 10.1186/s12889-016-3919-7, 28061761 PMC5217589

[64] Holm L Ekström MP Hach S Lund TB. Who is cooking dinner? Changes in the gendering of cooking from 1997 to 2012 in four Nordic countries. Food, Cult Soc. (2015) 18:589–610. doi: 10.1080/15528014.2015.1088191

[65] Emanuel AS McCully SN Gallagher KM Updegraff JA. Theory of planned behavior explains gender difference in fruit and vegetable consumption. Appetite. (2012) 59:693–7. doi: 10.1016/j.appet.2012.08.007, 22898607 PMC3490048

[66] Arganini C Saba A Comitato R Virgili F Turrini A. Gender differences in food choice and dietary intake in modern western societies. Public Health Soc Behav Health. (2012) 4:83–102. doi: 10.5772/37886

[67] Shepherd R. The psychology of food choice. Nutr Food Sci. (1990) 90:2–4. doi: 10.1108/eb059291

[68] Osaili TM Obaid RS Alkayyali SAI Ayman H Bunni SM Alkhaled SB . Consumers’ knowledge and attitudes about food additives in the UAE. PLoS One. (2023) 18:e0282495. doi: 10.1371/journal.pone.0282495, 36877679 PMC9987778

[69] AlBlooshi S Khalid A Hijazi R. The barriers to sustainable nutrition for sustainable health among Zayed university students in the UAE. Nutrients. (2022) 14:4175. doi: 10.3390/nu14194175, 36235827 PMC9571293

[70] Emirates News Agency - WAM President restructures social welfare programme of low-income citizens. Abu Dhabi, United Arab Emirates: Emirates News Agency (WAM). (2022).

[71] Khair H Bataineh MF Zaręba K Alawar S Maki S Sallam GS . Pregnant women’s perception and knowledge of the impact of obesity on prenatal outcomes—a cross-sectional study. Nutrients. (2023) 15:2420. doi: 10.3390/nu15112420, 37299384 PMC10254899

[72] Kamphuis CBM Giskes K de Bruijn G-J Wendel-Vos W Brug J Van Lenthe FJ. Environmental determinants of fruit and vegetable consumption among adults: a systematic review. Br J Nutr. (2006) 96:620–35. doi: 10.1079/BJN20061896 17010219

[73] Salas-García MA Bernal-Orozco MF Díaz-López A Betancourt-Núñez A Nava-Amante PA Vizmanos B. Associations of sociodemographic characteristics with food choice motives’ importance among Mexican adults: a cross-sectional analysis. Foods. (2025) 14:158. doi: 10.3390/foods14020158, 39856824 PMC11764962

[74] Aschemann-Witzel J Zielke S. Can't buy me green? A review of consumer perceptions of and behavior toward the price of organic food. J Consum Aff. (2017) 51:211–51.

[75] Fleming RM Chaudhuri TK. How expensive is it to lose weight?-the financial cost of one such weight loss program. J Cardiovasc Med Cardiol. (2020) 7:057–9. doi: 10.17352/2455-2976.000113

[76] Jeruszka-Bielak M Kollajtis-Dolowy A Santoro A Ostan R Berendsen AAM Jennings A . Are nutrition-related knowledge and attitudes reflected in lifestyle and health among elderly people? A study across five European countries. Front Physiol. (2018) 9:2018. doi: 10.3389/fphys.2018.00994, 30108512 PMC6079245

[77] Kubota Y Heiss G MacLehose RF Roetker NS Folsom AR. Association of educational attainment with lifetime risk of cardiovascular disease: the atherosclerosis risk in communities study. JAMA Intern Med. (2017) 177:1165–72. doi: 10.1001/jamainternmed.2017.1877, 28604921 PMC5710437

[78] Siu J Giskes K Turrell G. Socio-economic differences in weight-control behaviours and barriers to weight control. Public Health Nutr. (2011) 14:1768–78. doi: 10.1017/S1368980011000644, 21557867

[79] Bourdieu P. Distinction: A social critique of the judgement of taste In: Social stratification, class, race, and gender in sociological perspective. Second ed. Grusky D. B. London Country, United Kingdom: Routledge (2019). 499–525.

[80] Oude Groeniger J van Lenthe FJ Beenackers MA Kamphuis CBM. Does social distinction contribute to socioeconomic inequalities in diet: the case of ‘superfoods’ consumption. Int J Behav Nutr Phys Act. (2017) 14:1–7. doi: 10.1186/s12966-017-0495-x 28347301 PMC5369222

[81] Darko J Eggett DL Richards R. Shopping behaviors of low-income families during a 1-month period of time. J Nutr Educ Behav. (2013) 45:20–9. doi: 10.1016/j.jneb.2012.05.016, 23141001

[82] Webber CB Sobal J Dollahite JS. Shopping for fruits and vegetables. Food and retail qualities of importance to low-income households at the grocery store. Appetite. (2010) 54:297–303. doi: 10.1016/j.appet.2009.11.015, 19961886

[83] Robinson E Jones A Marty L. The role of health-based food choice motives in explaining the relationship between lower socioeconomic position and higher BMI in UK and US adults. Int J Obes. (2022) 46:1818–24. doi: 10.1038/s41366-022-01190-4, 35864310 PMC7613617

[84] Scalvedi ML Gennaro L Saba A Rossi L. Relationship between nutrition knowledge and dietary intake: an assessment among a sample of Italian adults. Front Nutr. (2021) 8:714493. doi: 10.3389/fnut.2021.714493, 34589511 PMC8473625

[85] Davy SR Benes BA Driskell JA. Sex differences in dieting trends, eating habits, and nutrition beliefs of a group of midwestern college students. J Am Diet Assoc. (2006) 106:1673–7. doi: 10.1016/j.jada.2006.07.017, 17000202

[86] Søgaard AJ Selmer R Bjertness E Thelle D. The Oslo health study: the impact of self-selection in a large, population-based survey. Int J Equity Health. (2004) 3:3. doi: 10.1186/1475-9276-3-3, 15128460 PMC428581

[87] UAE Ministry of Human Resources and Emiratisation Youth comprise over 51 per cent of UAE labour force. Dubai, United Arab Emirates: Al Nisr Publishing LLC, Gulf News. (2024). Available online at: https://gulfnews.com/uae/government/youth-comprise-over-51-per-cent-of-uae-labour-force-mohre-1.500014424

