# Supplementary material for: Exploring food choices among educated adults in the United Arab Emirates
Source: Front Nutr. 2026 Jan 12;12:1708017. doi: 10.3389/fnut.2025.1708017 (PMC12832438; doi:10.3389/fnut.2025.1708017)
Supplement: Supplementary file 1 [file Table_1.docx]

Supplementary Material

# Supplementary Tables

## Table 1: Data Collection Questionnaire (English language)

**Section 1: General information**

**General Information**

1. Are you male or female?
2. Male
3. Female
4. How old are you? ___________
5. Your height in cm __________
6. Your weight in kg __________
7. Your nationality __________
8. Are you:
9. Single
10. Married
11. What is your family size?
12. 1-2 members
13. 3-4 members
14. 5 or more members
15. What is the highest level of education you have completed?
16. Primary school
17. Secondary school
18. High school
19. Bachelor
20. Masters
21. Doctorate
22. Are you currently:
23. Employed full-time
24. Employed part-time
25. Unemployed
26. Retired
27. Student
28. What is your monthly income?
29. Less than 5000 AED
30. 5001 AED to 10,000 AED
31. 10,001 AED to 20,000 AED
32. 20,001 AED to 35,000 AED
33. 35,001 AED to 50,000 AED
34. Above 50,001 AED
35. Are you currently or have you ever consulted with a Nutritionist/Dietician?
36. Yes
37. No
38. Have you received any education and/or training about nutrition before?
39. Yes
40. No
41. Which of the following best describes your general familiarity with dietary guidelines about healthy diets?
42. I know a lot about them.
43. I know a fair amount about them.
44. I have heard about them but know very little about them.
45. I have never heard of them.

**Section 2: Food Choice Questionnaire**

| Item no. | It is important to me that the food I eat on a typical day: | Not important | A little important | Moderately important | Very important |
| --- | --- | --- | --- | --- | --- |
| 1 | Is easy to prepare |  |  |  |  |
| 2 | Is low in calories |  |  |  |  |
| 3 | Tastes good |  |  |  |  |
| 4 | Contains natural ingredients |  |  |  |  |
| 5 | Is not expensive |  |  |  |  |
| 6 | Is low in fat |  |  |  |  |
| 7 | Is familiar |  |  |  |  |
| 8 | Is high in fibre and roughage |  |  |  |  |
| 9 | Is easily available in shops and supermarkets |  |  |  |  |
| 10 | Is good value for money |  |  |  |  |
| 11 | Cheers me up |  |  |  |  |
| 12 | Smells nice |  |  |  |  |
| 13 | Can be cooked very simply |  |  |  |  |
| 14 | Helps me cope with stress |  |  |  |  |
| 15 | Helps me control my weight |  |  |  |  |
| 16 | Has a pleasant texture |  |  |  |  |
| 17 | Is packaged in an environmentally friendly way |  |  |  |  |
| 18 | Is like the food I ate when I was a child |  |  |  |  |
| 19 | Contains a lot of vitamins and minerals |  |  |  |  |
| 20 | Contains no artificial ingredients |  |  |  |  |
| 21 | Keeps me awake/alert |  |  |  |  |
| 22 | Looks nice |  |  |  |  |
| 23 | Helps me relax |  |  |  |  |
| 24 | Is high in protein |  |  |  |  |
| 25 | Takes no time to prepare |  |  |  |  |
| 26 | Keeps me healthy |  |  |  |  |
| 27 | Is good for my skin/teeth/hair/nails etc |  |  |  |  |
| 28 | Makes me feel good |  |  |  |  |
| 29 | Has the country of origin clearly marked |  |  |  |  |
| 30 | Is what I usually eat |  |  |  |  |
| 31 | Helps me to cope with life |  |  |  |  |
| 32 | Can be bought in shops close to where I live or work |  |  |  |  |
| 33 | Is cheap |  |  |  |  |
| 34 | Has been produced in a way that animals' rights have been respected |  |  |  |  |
| 35 | Has been produced in a way which has not shaken the balance of nature |  |  |  |  |
| 36 | Is organic |  |  |  |  |
| 37 | Has not been transported an excessive distance |  |  |  |  |

**Table 2: Data Collection Questionnaire (Arabic language)**

**القسم الأول: معلومات عامة**

1. **هل أنت ذكر أم أنثى**
2. ذكر
3. أنثى
4. **كم عمرك؟ ____________________**
5. **طولك (سم) ___________________**
6. **وزنك (كلغ) __________________**
7. **جنسيتك _____________________**
8. **هل أنت:**
9. أعزب / عازبة
10. منزوج / متزوجة
11. **كم هو عدد أفراد عائلتك؟**
12. 1 – 2 (فرد)
13. 3 – 4 (فرد)
14. 5 أفراد أو أكثر
15. **ما هو أعلى مستوى تعليمي قمت بتحصيله؟**
16. تعليم أساسي
17. المدرسة الابتدائية
18. المدرسة الثانوية
19. بكالوريوس
20. ماجستير
21. دكتوراه
22. **هل أنت حالياً:**
23. موظف / موظفة بدوام كامل
24. موظف / موظفة بدوام جزئي
25. عاطل / عاطلة عن العمل
26. متقاعد / متقاعدة
27. طالب / طالبة
28. **كم قيمة دخلك الشهري؟**
29. أقل من 5000 درهم إماراتي
30. 5001 درهم إماراتي حتى 10,000 درهم إماراتي
31. 10,001 درهم إماتي حتى 20,000 درهم إماراتي
32. 20,001 درهم إماراتي حتى 35,000 درهم إماراتي
33. 35,001 درهم إماراتي حتى 50,000 درهم إماراتي
34. 50,001 درهم إماراتي وما فوق
35. **هل تتبع حاليا نظام حمية غذائية أو هل سبق لك أن قمت باستشارة أخصائي تغذية؟**
36. نعم
37. لا
38. **هل حصلت على أي تعليمات و / أو تدريبات حول أي نظام تغذية من قبل؟**
39. نعم
40. لا
41. **أي من الخيارات يحدد بشكل أفضل معرفتك العامة بالإرشادات الغذائية المتعلقة بالأنظمة الغذائية الصحية؟**
42. أعرف الكثير عنها
43. أعرف قدرًا لا بأس به عنها.
44. لقد سمعت عنها ولكني أعرف القليل جدًا عنها.
45. لم اسمع بها أبدا

**القسم الثاني: استبيان حول اختيار الطعام**

| **رقم الصنف الجديد** | **الأمر المهم بالنسبة لي أن الطعام الذي أتناوله في يوم المعتاد:** | **غير هام** | **قليل الأهمية** | **متوسط الأهمية** | **بالغ الأهمية** |
| --- | --- | --- | --- | --- | --- |
| **1** | **سهل التحضير** |  |  |  |  |
| **2** | **منخفضة السعرات الحرارية** |  |  |  |  |
| **3** | **جيد المذاق** |  |  |  |  |
| **4** | **يحتوي على مكونات طبيعية** |  |  |  |  |
| **5** | **غير باهظ الثمن** |  |  |  |  |
| **6** | **منخفض الدهون** |  |  |  |  |
| **7** | **طعام شائع** |  |  |  |  |
| **8** | **غني بالألياف والأعشاب** |  |  |  |  |
| **9** | **متوفر بسهولة في المحلات التجارية ومحلات السوبر ماركت** |  |  |  |  |
| **10** | **ذات سعر مقبول** |  |  |  |  |
| **11** | **يرفع من مستوى سعادتي** |  |  |  |  |
| **12** | **ذات رائحة شهية** |  |  |  |  |
| **13** | **يمكن طهيه بطريقة بسيطة** |  |  |  |  |
| **14** | **يساعدني في التغلب على التوتر** |  |  |  |  |
| **15** | **يساعدني في التحكم في وزني** |  |  |  |  |
| **16** | **ذات مظهر رائع ولذيذ** |  |  |  |  |
| **17** | **مُعبأ بطريقة صديقة للبيئة** |  |  |  |  |
| **18** | **مثل الطعام الذي أكلته عندما كنت طفلاً** |  |  |  |  |
| **19** | **يحتوي على الكثير من الفيتامينات والمعادن** |  |  |  |  |
| **20** | **لا يحتوي على مكونات صناعية** |  |  |  |  |
| **21** | **يبقيني مستيقظا / متنبها** |  |  |  |  |
| **22** | **يبدو لذيذا** |  |  |  |  |
| **23** | **يساعدني على الاسترخاء** |  |  |  |  |
| **24** | **غني بالبروتين** |  |  |  |  |
| **25** | **لا يستغرق وقتا طويلا للتحضير** |  |  |  |  |
| **26** | **يبقيني بصحة جيدة** |  |  |  |  |
| **27** | **مفيد لبشرتي / أسناني / شعري / أظافري، الخ.** |  |  |  |  |
| **28** | **يمنحني شعور بصحة جيدة** |  |  |  |  |
| **29** | **تكون علامة بلد المنشأ واضحة** |  |  |  |  |
| **30** | **طعام أتناوله عادة** |  |  |  |  |
| **31** | **يساعدني على التأقلم مع الحياة** |  |  |  |  |
| **32** | **يمكن شراؤها من المحلات القريبة من محل إقامتي أو عملي** |  |  |  |  |
| **33** | **رخيص الثمن** |  |  |  |  |
| **34** | **تم إنتاجه بطريقة تحفظ حقوق الحيوانات** |  |  |  |  |
| **35** | **تم إنتاجه بطريقة لا تخل بتوازن الطبيعة** |  |  |  |  |
| **36** | **عضوي** |  |  |  |  |
| **37** | **غير منقول من مسافة بعيدة** |  |  |  |  |

**Table 3: Variance Inflation Factor (VIF) and Tolerance Values for Predictors Included in the Multivariable Regression Models**

| Predictors | | Collinearity Statistics | |
| --- | --- | --- | --- |
|  |  | Tolerance | VIF |
| Age |  | 0.349 | 2.866 |
| Sex | Female | 0.887 | 1.128 |
|  | Male (ref.) |  |  |
| Nationality | UAE national | 0.646 | 1.548 |
|  | Expatriate resident (ref.) |  |  |
| Marital status | Single | 0.553 | 1.808 |
|  | Married (ref.) |  |  |
| Family size | 4 or less members (ref.) |  |  |
|  | 5 or more members | 0.759 | 1.317 |
| Educational level | High school | 0.236 | 4.241 |
|  | Undergraduate | 0.3 | 3.328 |
|  | Postgraduate (ref.) |  |  |
| Employment status | Unemployed/student | 0.58 | 1.724 |
|  | Employed (ref.) |  |  |
| Monthly income, AED | Less than 5000 | 0.559 | 1.79 |
|  | 5001 – 20000 | 0.549 | 1.82 |
|  | More than 20000 (ref.) |  |  |
| Diet counseling | No | 0.757 | 1.321 |
|  | Yes (ref.) |  |  |
| Nutrition education | No | 0.74 | 1.352 |
|  | Yes (ref.) |  |  |
| Knowledge of dietary guidelines | I have never heard about them (ref.) |  |  |
|  | I have heard about them but know very little | 0.203 | 4.921 |
|  | I know a fair amount | 0.153 | 6.522 |
|  | I know a lot | 0.205 | 4.867 |
| BMI |  | 0.93 | 1.076 |

**Table 4: Participant characteristics by gender**

| Variable | | Total (*n*=894) | Males (*n*=202) | Females (*n*=692) | ES |
| --- | --- | --- | --- | --- | --- |
| Age | 18 – 25 years old | 695 (77.7) | 153 (75.7) | 542 (78.3) | 0.10 |
|  | 26 – 45 years old | 187 (20.9) | 42 (20.8) | 145 (21.0) |  |
|  | >45 years old | 12 (1.3) | 7 (3.5) | 5 (0.7) |  |
| Nationality | UAE national | 590 (66.0) | 81 (40.1) | 223 (32.2) | 0.07 |
|  | Expatriate resident | 304 (34.0) | 121 (59.9) | 469 (67.8) |  |
| Marital status | Single | 798 (89.3) | 32 (15.8) | 64 (9.2) | 0.09 |
|  | Married | 96 (10.7) | 170 (84.2) | 628 (90.8) |  |
| Family size | 4 or less members | 180 (20.1) | 51 (25.2) | 129 (18.6) | 0.07 |
|  | 5 or more members | 714 (79.9) | 151 (74.8) | 563 (81.4) |  |
| Educational level | High school | 440 (49.2) | 117 (57.9) | 323 (46.7) | 0.11 |
|  | Undergraduate | 291 (32.6) | 48 (23.8) | 243 (35.1) |  |
|  | Postgraduate | 163 (18.2) | 37 (18.3) | 126 (18.2) |  |
| Employment status | Unemployed/student | 132 (85.2) | 46 (22.8) | 86 (12.4) | 0.12 |
|  | Employed | 762 (14.8) | 156 (77.2) | 606 (87.6) |  |
| Monthly income, AED | Less than 5000 | 503 (56.3) | 89 (44.1) | 414 (59.8) | 0.13 |
|  | 5001 –20000 | 206 (23.0) | 60 (29.7) | 146 (21.1) |  |
|  | More than 20000 | 185 (20.7) | 53 (26.2) | 132 (19.1) |  |
| Diet counseling | No | 616 (68.9) | 150 (74.3) | 466 (67.3) | –– |
|  | Yes | 278 (31.1) | 52 (25.7) | 226 (32.7) |  |
| Nutrition education | No | 479 (53.6) | 131 (64.9) | 348 (50.3) | 0.12 |
|  | Yes | 415 (46.4) | 71 (35.1) | 344 (49.7) |  |
| Knowledge of dietary guidelines | I have never heard about them | 39 (4.4) | 19 (9.4) | 20 (2.9) | 0.17 |
|  | I have heard about them but know very little | 199 (22.3) | 59 (29.2) | 140 (20.2) |  |
|  | I know a fair amount | 479 (53.6) | 95 (47.0) | 384 (55.5) |  |
|  | I know a lot | 177 (19.8) | 29 (14.4) | 148 (21.4) |  |
| BMI categories | Underweight | 107 (12.0) | 14 (6.9) | 93 (13.4) | 0.14 |
|  | Normal weight | 422 (47.2) | 84 (41.6) | 338 (48.8) |  |
|  | Overweight | 221 (24.7) | 57 (28.2) | 164 (23.7) |  |
|  | Obesity | 144 (16.1) | 47 (23.3) | 97 (14.0) |  |

ES: effect size. All the data in the table are presented as n(%). Significance set as *p*-value ≤ 0.05.

# Supplementary Figures

**Figure 1. Normal P–P Plots for All Nine Models**

| 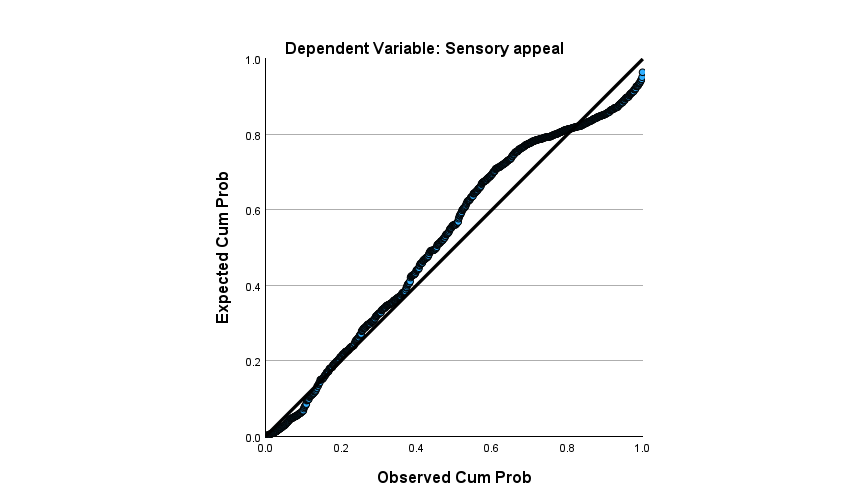 | 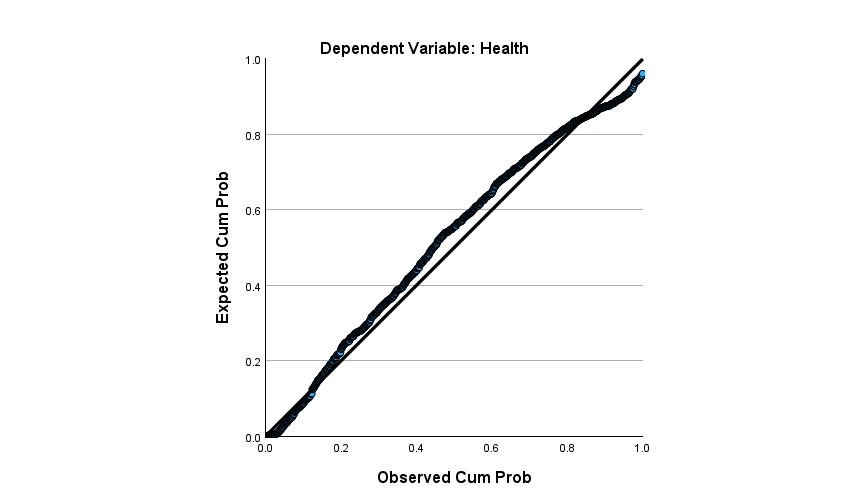 |
| --- | --- |
| 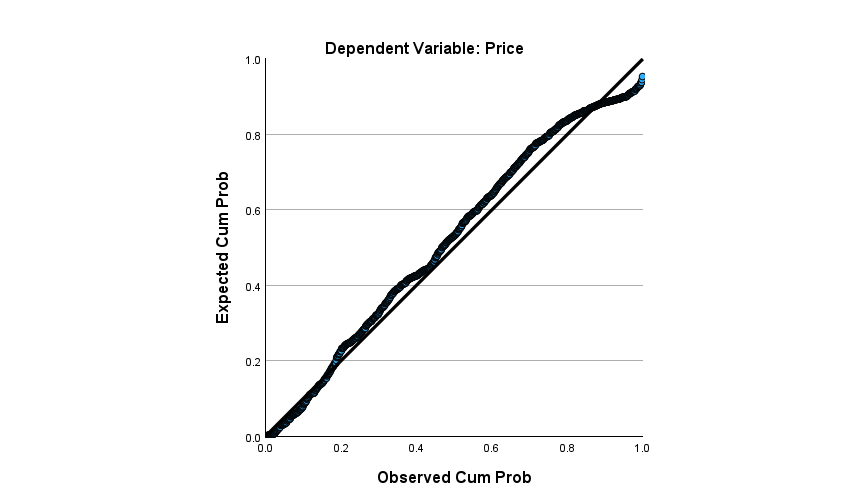 | 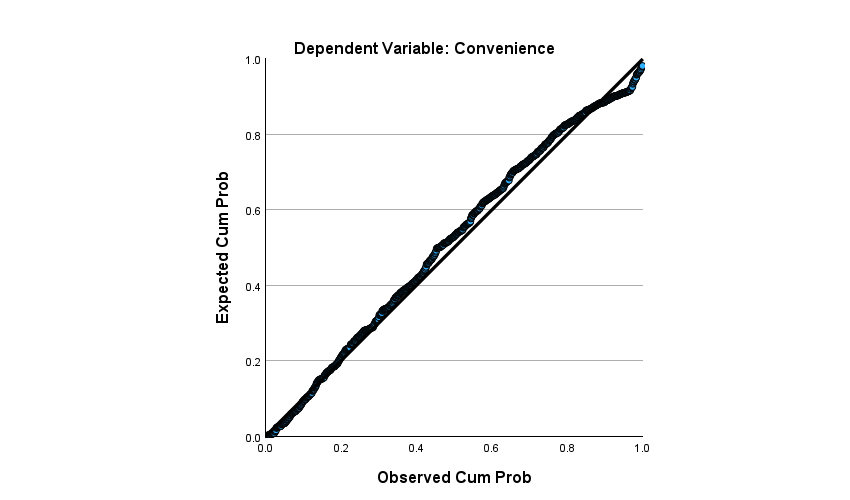 |
| 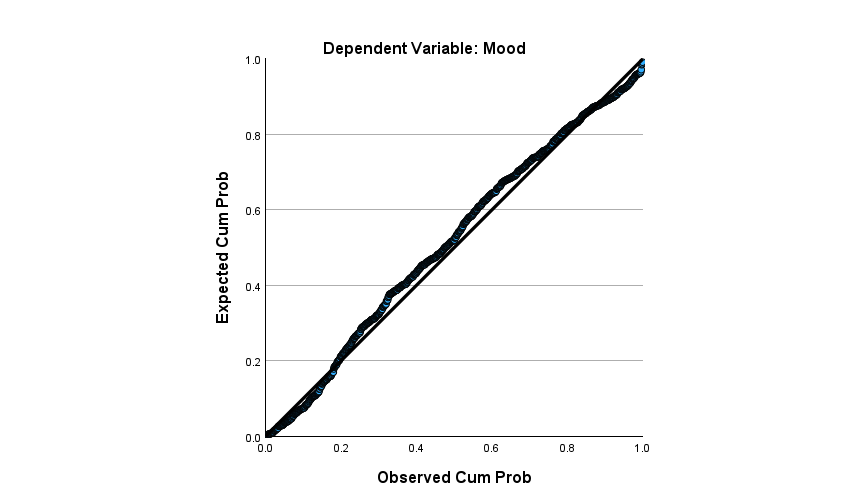 | 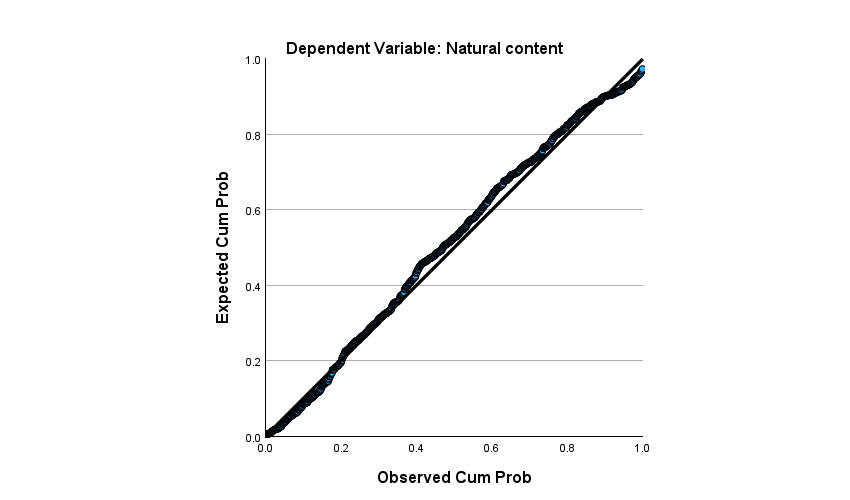 |
| 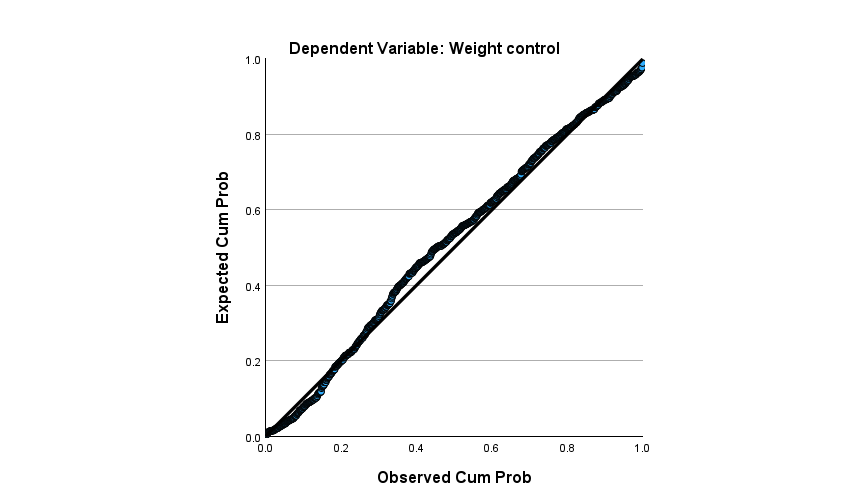 | 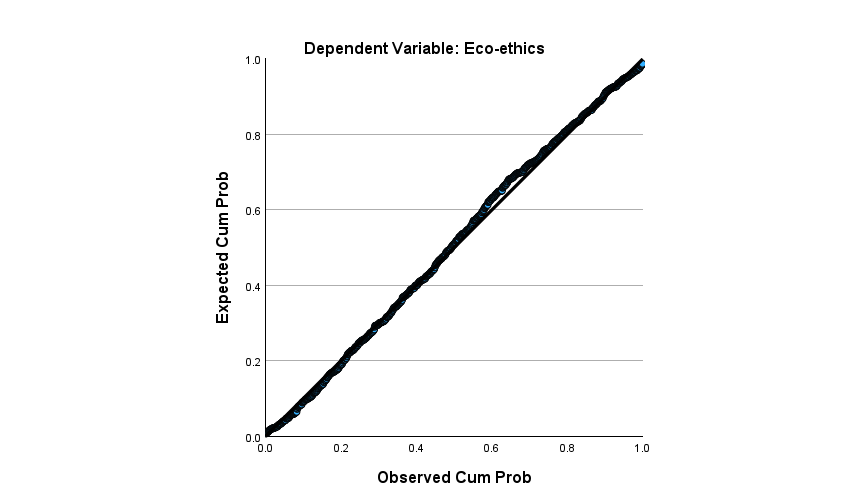 |
| 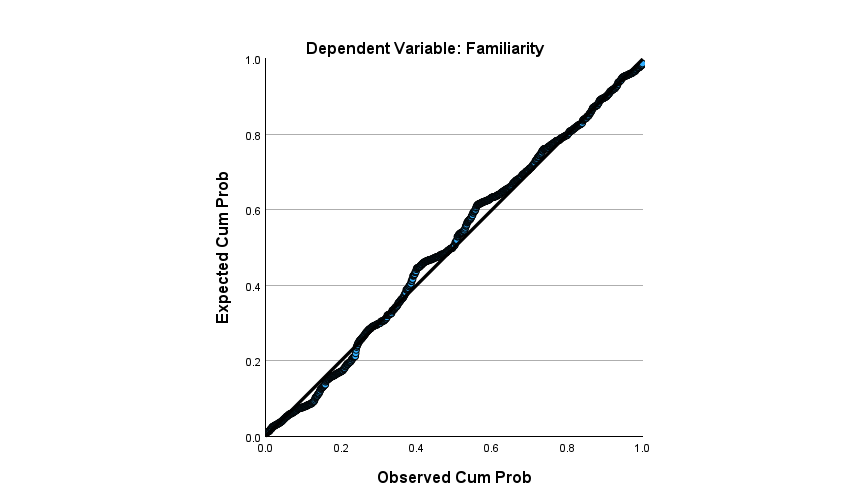 |  |

**Figure 2. Normal P–P Plots for All Nine Models**

| 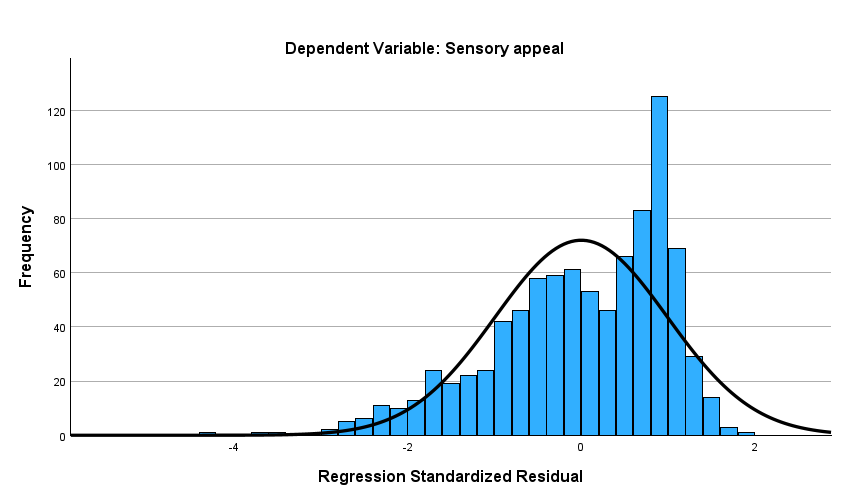 | 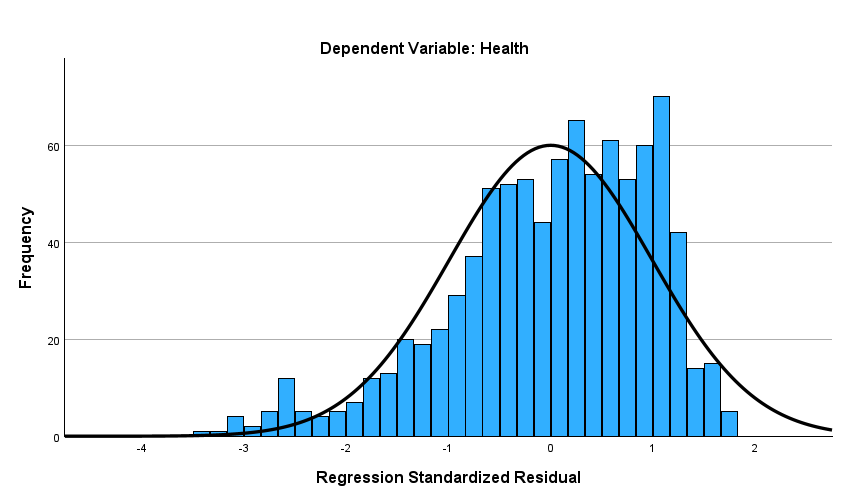 |
| --- | --- |
| 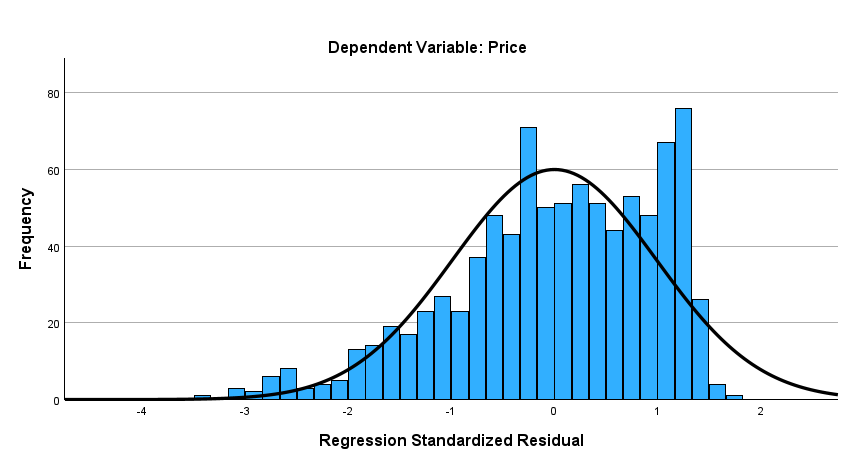 | 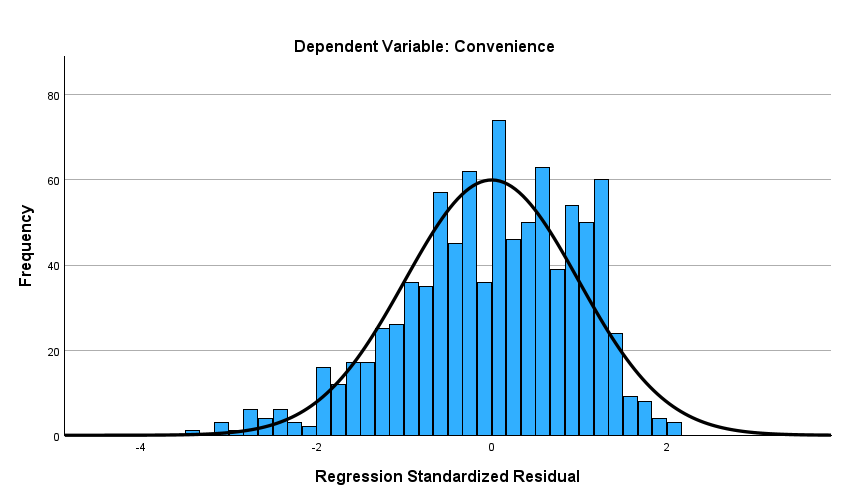 |
| 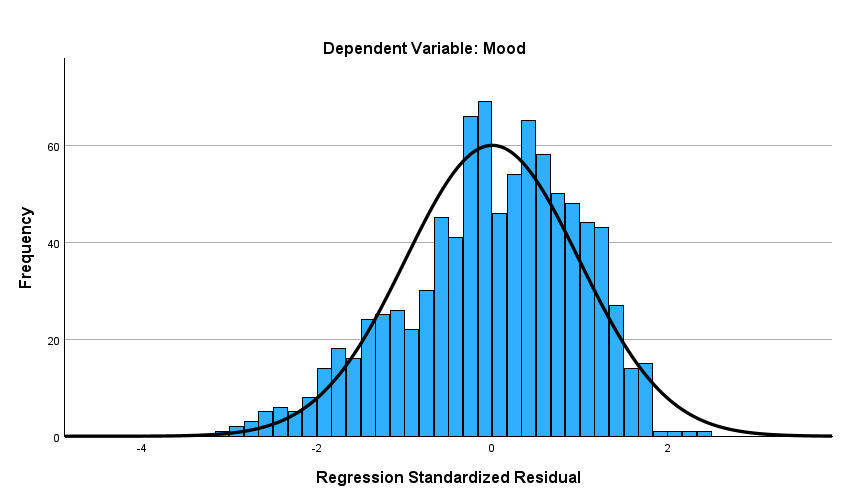 | 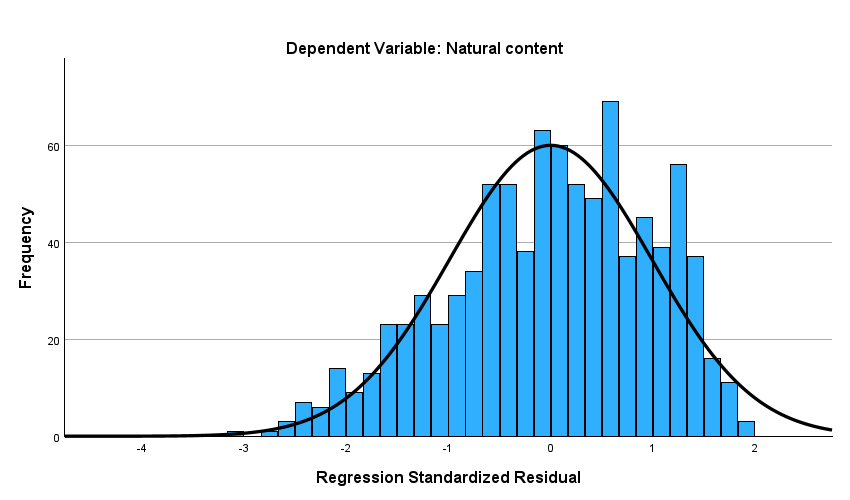 |
| 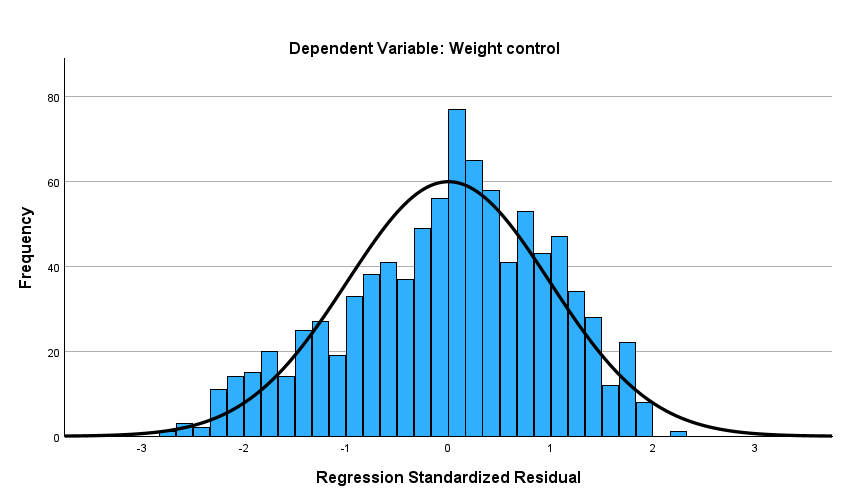 | 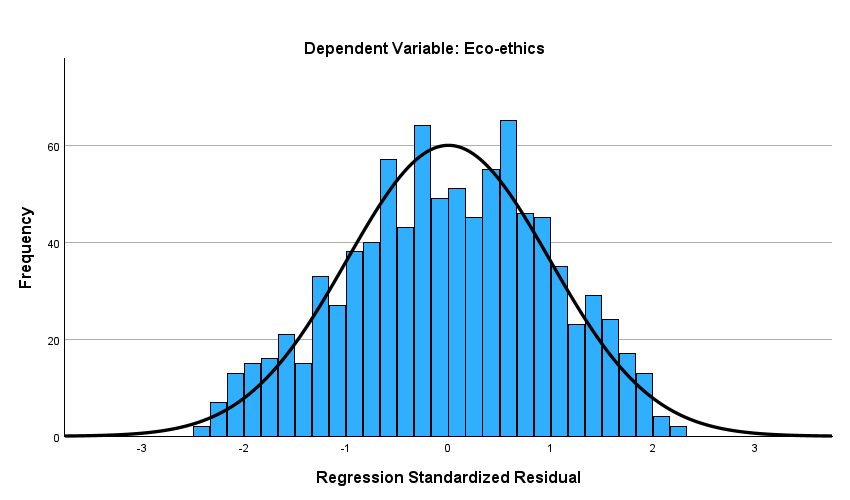 |
| 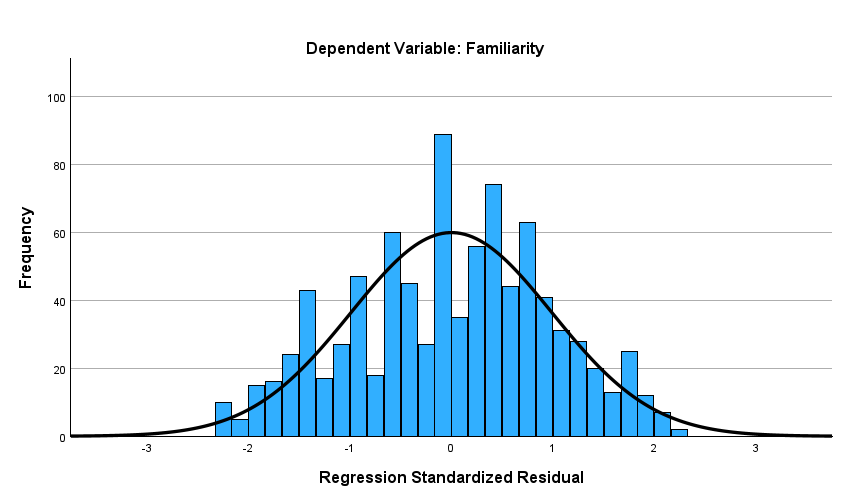 |  |

| 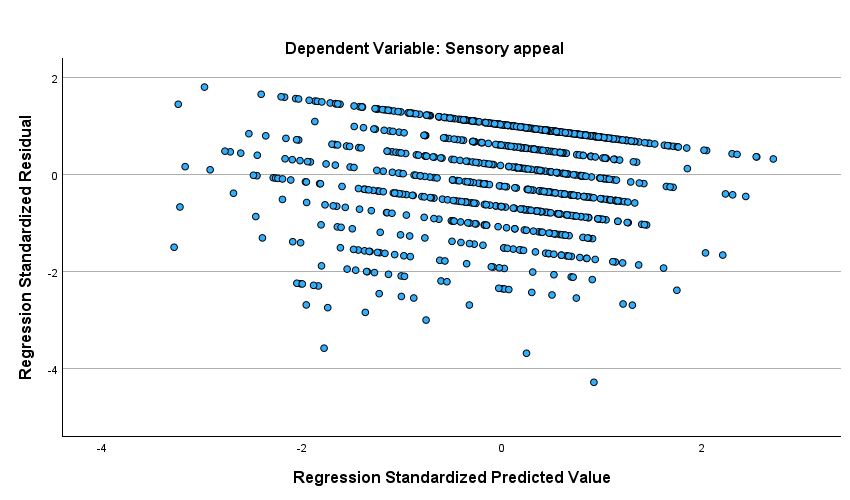 | 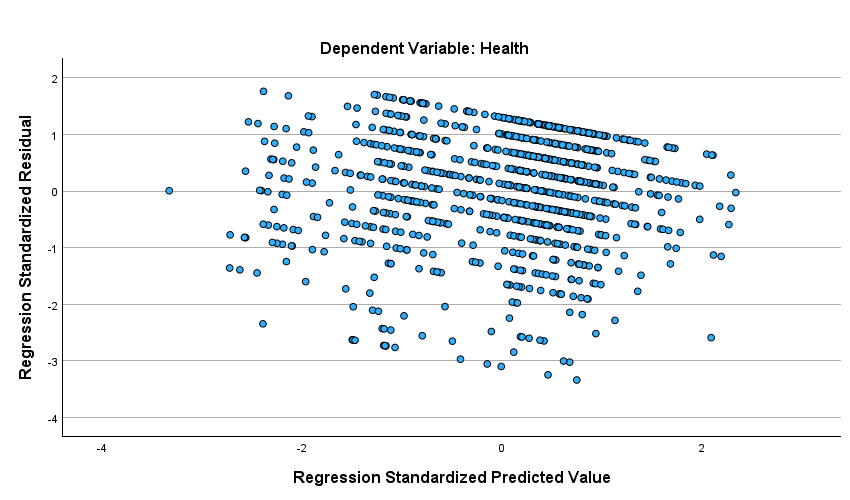 |
| --- | --- |
| 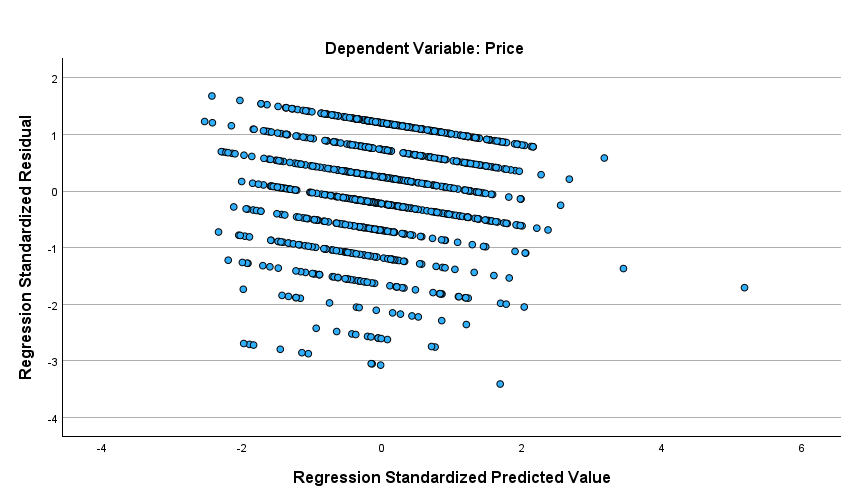 | 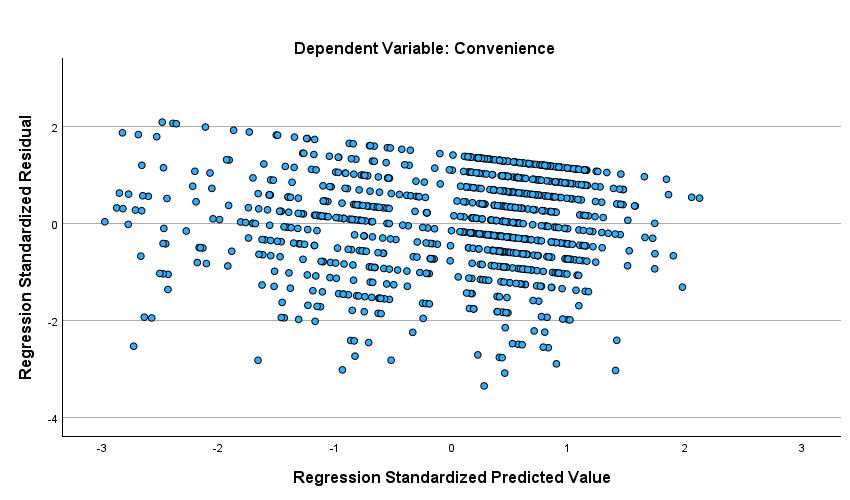 |
| 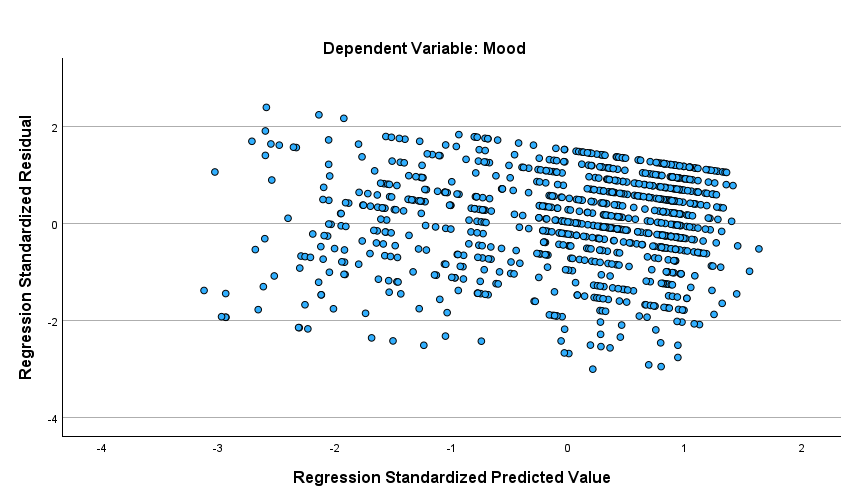 | 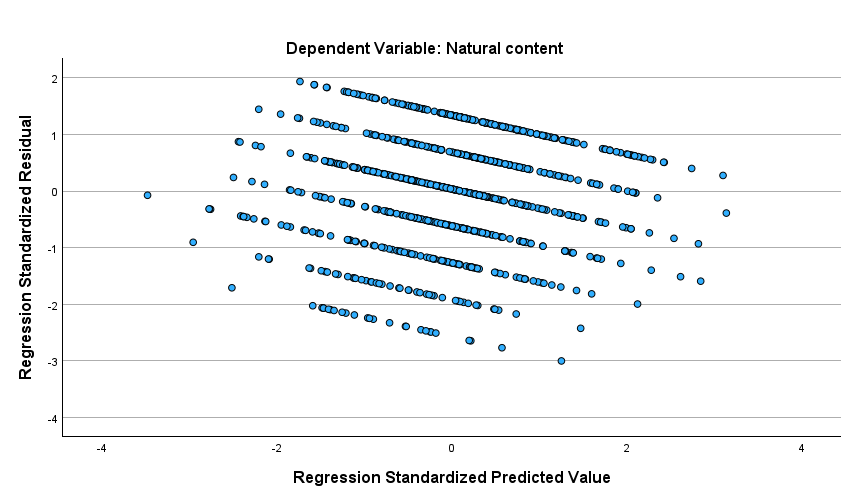 |
| 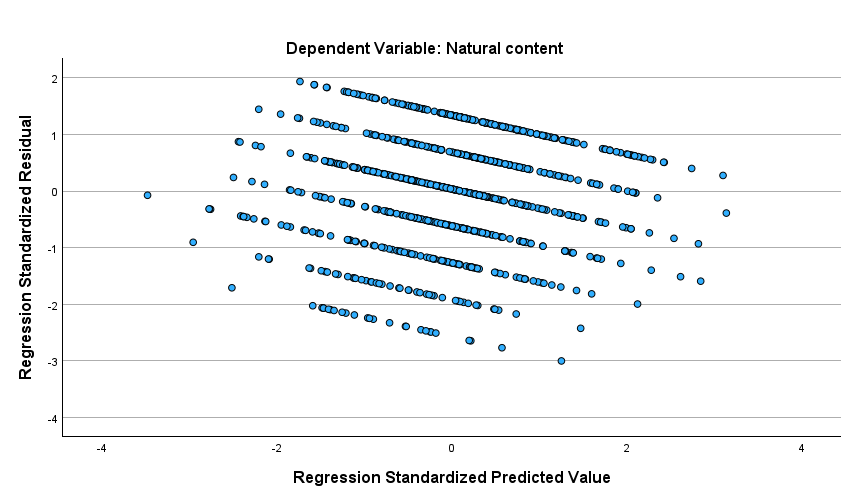 | 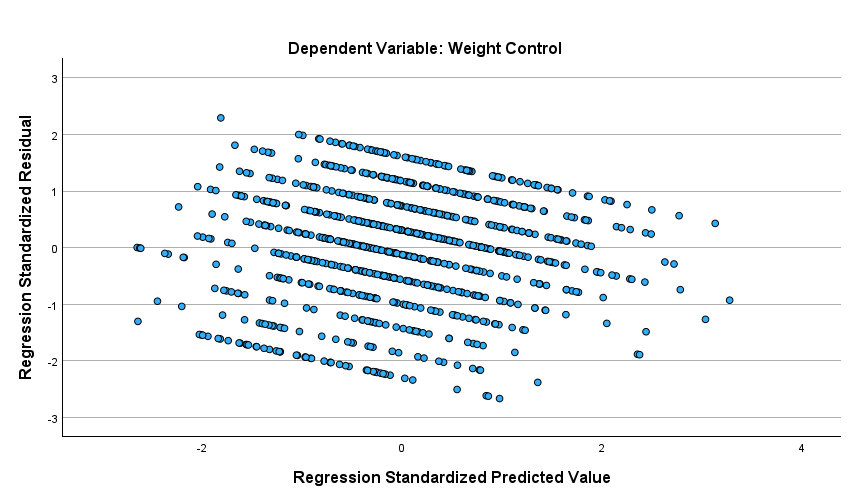 |
| 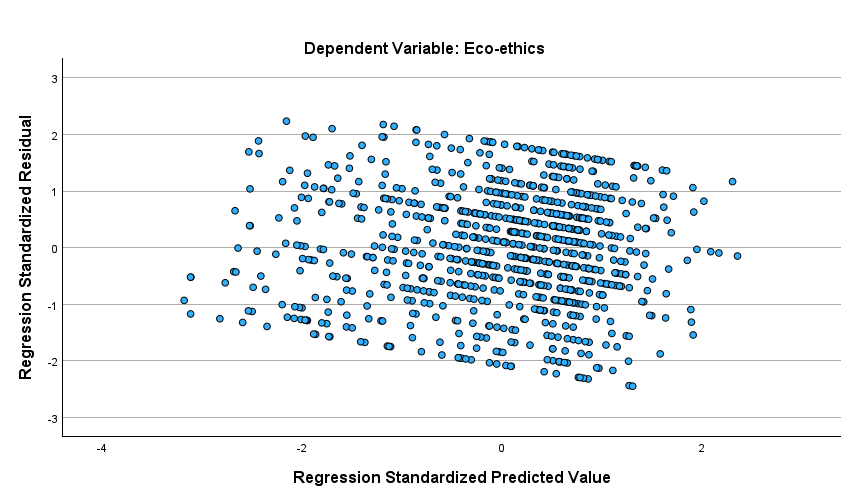 | 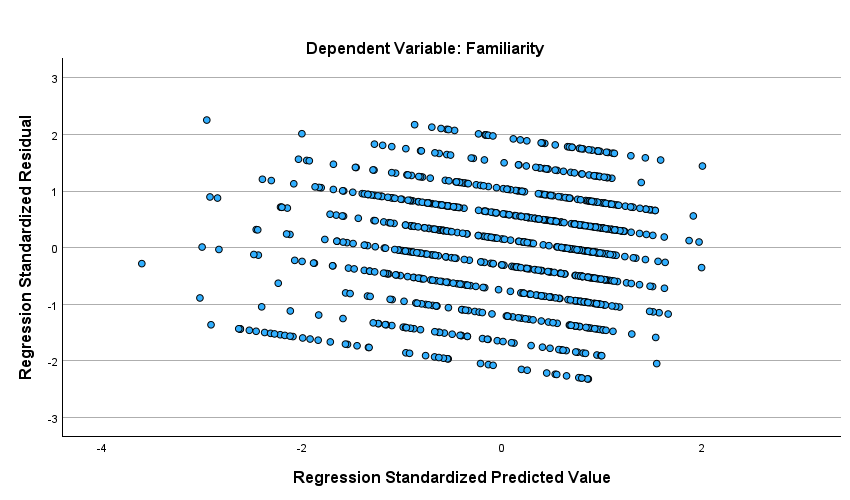 |
